# IL-36**γ** drives skin toxicity induced by EGFR/MEK inhibition and commensal *Cutibacterium acnes*

**DOI:** 10.1172/JCI128678

**Published:** 2020-02-04

**Authors:** Takashi K. Satoh, Mark Mellett, Barbara Meier-Schiesser, Gabriele Fenini, Atsushi Otsuka, Hans-Dietmar Beer, Tamara Rordorf, Julia-Tatjana Maul, Jürg Hafner, Alexander A. Navarini, Emmanuel Contassot, Lars E. French

**Affiliations:** 1 Department of Dermatology, University of Zürich, Zürich, Switzerland.; 2 Department of Dermatology, Kyoto University, Kyoto, Japan.; 3 Medical Faculty, University of Zürich, Zürich, Switzerland.; 4 Clinic for Oncology, University Hospital Zürich, Zürich, Switzerland.; 5 Department of Dermatology, University Hospital of Basel, Basel, Switzerland.; 6 Department of Dermatology and Allergology, Ludwig Maximilian University of Munich, Munich, Germany.

**Keywords:** Dermatology, Inflammation, Cytokines, Molecular biology, Skin

## Abstract

Epidermal growth factor receptor (EGFR) and MEK inhibitors (EGFRi/MEKi) are beneficial for the treatment of solid cancers but are frequently associated with severe therapy-limiting acneiform skin toxicities. The underlying molecular mechanisms are poorly understood. Using gene expression profiling we identified IL-36γ and IL-8 as candidate drivers of EGFRi/MEKi skin toxicity. We provide molecular and translational evidence that EGFRi/MEKi in concert with the skin commensal bacterium *Cutibacterium acnes* act synergistically to induce IL-36γ in keratinocytes and subsequently IL-8, leading to cutaneous neutrophilia. IL-36γ expression was the combined result of *C*. *acnes*–induced NF-κB activation and EGFRi/MEKi–mediated expression of the transcription factor Krüppel-like factor 4 (KLF4), due to the presence of both NF-κB and KLF4 binding sites in the human IL-36γ gene promoter. EGFRi/MEKi increased KLF4 expression by blockade of the EGFR/MEK/ERK pathway. These results provide an insight into understanding the pathological mechanism of the acneiform skin toxicities induced by EGFRi/MEKi and identify IL-36γ and the transcription factor KLF4 as potential therapeutic targets.

## Graphical abstract

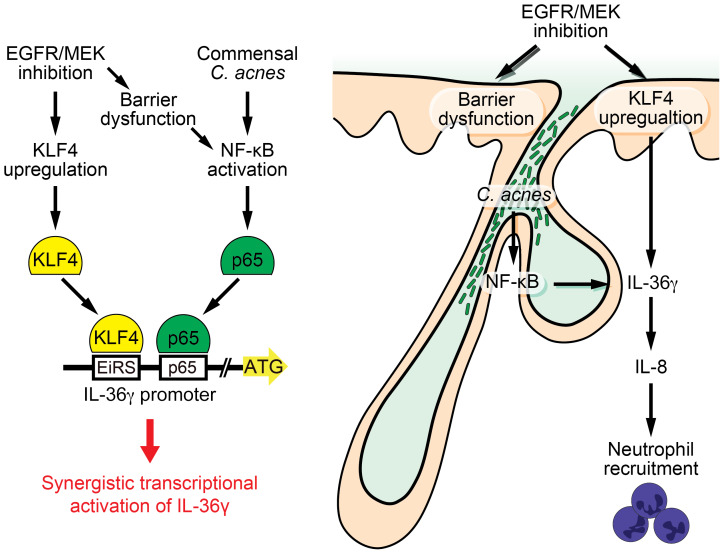

## Introduction

Agents targeting the epidermal growth factor receptor–mediated (EGFR-mediated) signaling pathway are increasingly used for the treatment of advanced lung, pancreatic, colorectal, and head and neck cancers, which benefit from exacerbated EGFR activity for their growth and survival (1, 2). Small-molecule inhibitors of the mitogen-activated protein kinase (MAPK) signaling pathways including extracellular signal–regulated kinase (ERK) and MAPK/ERK kinase 1 and 2 (MEK1 and -2) have also shown their efficacy in the treatment of various cancers, especially melanoma (3, 4).

One significant clinical limitation to the prolonged use of EGFR and MEK inhibitors (EGFRi/MEKi) is the occurrence of skin toxicities in 50%–80% of patients, including an acneiform eruption that usually develops within the first few weeks of therapy (4–6). The first monoclonal antibody and small-molecule inhibitor of EGFR were approved by the FDA for the treatment of cancer 12 and 13 years ago, respectively, and since then skin toxicities induced by these drugs remain unsolved problems. Even though topical or oral agents alone or in combination are used to treat skin toxicities and show some efficacy, EGFRi/MEKi–induced skin acneiform eruptions can still seriously affect patients’ quality of life, leading physicians to reduce the dose administered or discontinue therapy in severe skin toxicity cases (7–9). Importantly, the development and severity of the acneiform eruption have been shown to correlate with favorable antitumor responses (10–12).

The exact molecular pathogenesis underlying the frequent and rapid development of EGFRi/MEKi toxicity to skin is not understood to date. Animal studies using mice selectively lacking EGFR in the skin revealed that EGFR signaling is critical for normal skin barrier function and antimicrobial defense (13, 14). However, the phenotype of mice selectively lacking EGFR in the skin resembles atopic dermatitis and is distinct from the acneiform skin toxicity seen in patients treated with EGFRi/MEKi (15, 16). In humans, histopathology of acneiform eruption lesions is characterized by folliculitis with massive infiltration of neutrophils histologically resembling acne vulgaris (5). Another feature of acneiform toxicities caused by EGFRi/MEKi and shared with acne vulgaris is the topographical predominance of inflammation in skin areas rich in pilosebaceous units, also corresponding to sebum-rich regions of the skin, such as the central face, upper chest, and back (17–21). These sebum-rich regions are highly colonized by *Cutibacterium acnes* (formerly known as *Propionibacterium acnes*), a lipophilic commensal representing the most abundant microorganism on the skin of healthy adults (19–22). Although *C*. *acnes* is thought to play an important role in common acne, its involvement in EGFRi/MEKi acneiform toxicities has never been investigated to the best of our knowledge.

A better understanding of the molecular pathogenesis of acneiform eruption caused by EGFRi/MEKi is still needed so as to guide the development of effective therapies to prevent or suppress the skin toxicity, while preserving their antitumor effects. Here, we investigate the molecular mechanisms of acneiform eruption associated with EGFRi/MEKi.

## Results

### Skin gene expression profiling in EGFRi-induced acneiform skin toxicity.

**Figure 1:**
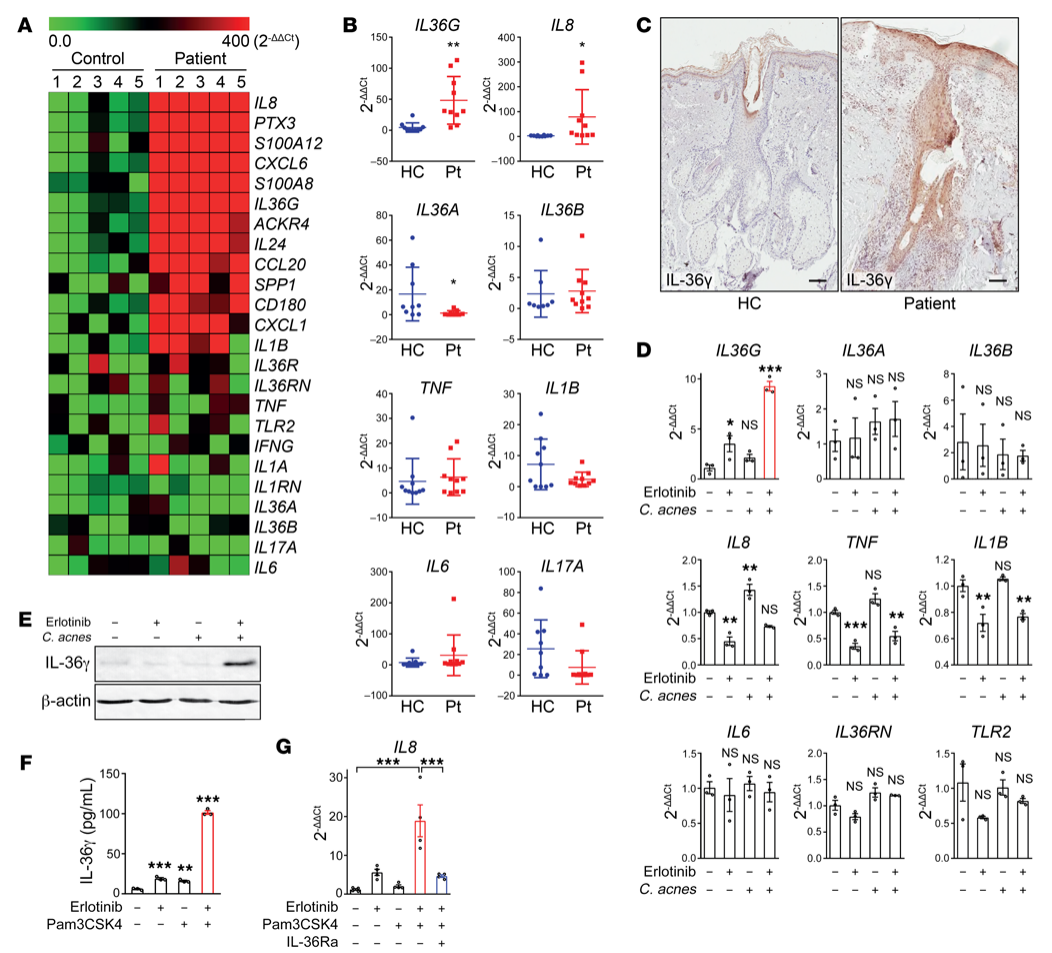
Increased production of IL-36γ in primary keratinocytes and lesional skin of patients suffering from acneiform eruptions in response to EGFR inhibition and *C. acnes*. (**A**) Gene expression profiling from lesional skin of 5 patients and 5 healthy controls (HC). Heatmap of the top 12 most differentially expressed genes ranked from lowest false discovery rate (FDR) and 12 selected genes are shown. (**B**) Quantitative PCR (qPCR) of mRNA from lesional skin samples of 10 EGFR inhibitor–treated patients with acneiform eruption and 10 healthy control skin biopsies. Data represent mean ± SD. (**C**) Immunohistochemical staining with goat anti–IL-36γ antibody of formalin-fixed, paraffin-embedded skin sections of acneiform eruption patient and normal donors. Scale bars: 100 μm. Pictures are representative of 5 patients and 5 healthy individuals. (**D**) PHKs were exposed to erlotinib (EGFR inhibitor, 1 μM) and *C*. *acnes* (MOI of 10) for 6 hours. Total RNA was analyzed by qPCR. Data represent mean ± SEM (*n* = 3). (**E**) PHKs were exposed to erlotinib (1 μM) or *C*. *acnes* (MOI of 10) or both for 24 hours. Cell lysates were analyzed by Western blotting using specific antibodies against IL-36γ and β-actin. Blots were run contemporaneously with the same protein samples. (**F**) PHKs were exposed to erlotinib (1 μM) and Pam3CSK4 (5 μg/mL). IL-36γ secretion was measured by ELISA in culture supernatants. Data represent mean ± SEM (*n* = 3). (**G**) Ex vivo skin explants were exposed to erlotinib (1 μM), Pam3CSK4 (5 μg/mL), and/or human IL-36Ra (1 μg/mL). The skin samples were then analyzed by qPCR. Data represent mean ± SEM (*n* = 4). Data were analyzed with 2-tailed unpaired *t* test (**B**), and 1-way ANOVA followed by Dunnett’s (**D** and **F**) or Tukey’s multiple-comparisons test (**G**). *P < 0.05; **P < 0.01; ***P < 0.001. Data are representative of 3 independent experiments.

Employing an unbiased approach, we performed gene expression profiling of lesional skin biopsy samples from patients suffering from acneiform eruption caused by EGFRi (Figure 1A and Supplemental Table 1; supplemental material available online with this article; https://doi.org/10.1172/JCI128678DS1). We found elevated IL-8 and IL-36γ in the patients’ skin, whereas important inflammatory cytokines such as TNF-α, IL-6, and IL-17A were not significantly upregulated when compared to skin from healthy donors (Figure 1A). This observation was further confirmed by quantitative PCR with more lesional skin samples (Figure 1B and Supplemental Figure 1A). As previously reported, the expression of antimicrobial peptides such as RNase7 was also found to be decreased in patients’ skin (ref. 14 and Supplemental Figure 1A). IL-36γ is a proinflammatory cytokine of the IL-1 family, predominantly expressed by keratinocytes and is able to signal in an auto- or paracrine manner through the IL-36 receptor (also known as IL1RL2) and activates the NF-κB signaling pathway in target cells. It has recently been shown that IL-36 plays a role in the cutaneous neutrophilic pustular autoinflammatory disease called DITRA (deficiency of the IL-36 receptor antagonist) (23, 24). Interestingly, IL-36γ has been demonstrated to induce prominent production of the potent neutrophil chemoattractant IL-8 (25), which would be compatible with the extensive infiltration of neutrophils seen in skin lesions from patients suffering from acneiform eruptions (5). Furthermore, clinical trial data have shown that subcutaneous anti–IL-8 antibody injection strongly abrogates the induction of acneiform skin toxicity by EGFRi (26). To define the cell types expressing IL-36γ in the skin of patients with acneiform eruption, immunohistochemical analyses and mRNA in situ hybridization were performed. In line with gene expression data, histochemical analysis of patients’ lesions revealed elevated IL-36γ expression, which was predominantly localized in keratinocytes of epidermal hair follicles (Figure 1C and Supplemental Figure 1, B and C). This result and the fact that EGFR is preferentially expressed in undifferentiated and proliferating keratinocytes in the basal and suprabasal layers of the epidermis as well as the outer layers of the hair follicle (5) led to the hypothesis that keratinocytes might be key players in the acneiform eruption by producing IL-36γ in response to EGFRi.

### EGFRi and C. acnes synergize to promote IL-36γ expression and skin inflammation.

To examine whether EGFR inhibition could lead to enhanced IL-36γ production in keratinocytes, primary human keratinocytes (PHKs) were exposed to the EGFRi erlotinib in vitro. Upon exposure to 1 μM erlotinib — a concentration compatible with the serum concentration found in treated patients (27) — PHKs produced 3.2-fold (*P* = 0.048) higher levels of IL-36γ than upon exposure to vehicle alone as quantified by quantitative PCR (Figure 1D). Given that both common acne vulgaris and EGFRi-induced eruptions occur in sebum-rich regions of the body that are colonized with *C*. *acnes,* and that *C*. *acnes* is known to be involved in the pathogenesis of acne (19–22), we exposed PHKs to both erlotinib and *C*. *acnes*. Interestingly, IL-36γ production at the mRNA and protein level was further enhanced (8.4-fold in mRNA, *P* = 0.001) when PHKs were simultaneously exposed to erlotinib and *C*. *acnes* (Figure 1, D and E). In contrast, the transcripts of other inflammatory cytokines such as TNF-α, IL-1β, and IL-6 were not significantly increased by simultaneous exposure to erlotinib and *C*. *acnes* (Figure 1D). Similar levels of IL-36γ induction in PHKs were also observed with cetuximab, another EGFRi, when used in combination with *C*. *acnes* (Supplemental Figure 1D). Furthermore, these results were confirmed when EGFR was genetically silenced using siRNA (Supplemental Figure 1E).

Besides IL-36γ, expression of other genes was significantly increased in acneiform lesions by EGFR inhibition, including the S100 proteins S100A12 and S100A8, the chemokine CXCL6, and the pleiotropic immunomodulatory cytokine IL-24 (Figure 1A). The regulation of the expression of these genes by erlotinib and *C*. *acnes* was also assessed in PHKs, and with the exception of S100A8, the expression of these genes was not as elevated as that of IL-36γ (Supplemental Figure 1F). However, the expression of the above transcripts could be significantly induced in PHKs by exposure for 6 hours to IL-36γ alone (Supplemental Figure 1G), suggesting that IL-36γ may be an upstream driver cytokine in EGFRi-induced acneiform eruption.

Similarly to exposure to the TLR2 agonist *C*. *acnes* (28–30), IL-36γ release into the culture supernatant of PHKs could be induced by exposure to erlotinib and the TLR2 agonist Pam3CSK4 (Figure 1F). In line with this, knocking down TLR2 attenuated IL-36γ production induced by erlotinib and *C*. *acnes* (Supplemental Figure 1, H and I). Expression of the neutrophil chemoattractant IL-8 has previously been shown to be induced by IL-36γ (23, 25, 31–35) and was also found by gene expression profiling and quantitative PCR to be upregulated (32-fold, *P* = 0.041) in acneiform lesional skin (Figure 1B). To determine if simultaneous EGFR inhibition and TLR2 signaling can trigger IL-36γ–dependent production of IL-8 in human skin, we exposed normal human skin ex vivo to erlotinib and Pam3CSK4. In line with the neutrophil-rich inflammation and enhanced IL-8 gene expression observed in acneiform lesional skin by EGFR inhibition, increased IL-8 production (16-fold, *P* = 0.0051) was observed in human skin explants exposed to erlotinib and Pam3CSK4 as compared with vehicle, erlotinib, or Pam3CSK4 alone (Figure 1G). In the same ex vivo experimental setting, addition of the recombinant IL-36 receptor antagonist (IL-36Ra) to erlotinib and Pam3CSK4 resulted in a significant reduction of IL-8 production (4.1-fold, *P* = 0.014) (Figure 1G), thus establishing the IL-36 dependency of IL-8 expression in human skin exposed simultaneously to EGFRi and TLR2 agonists. These data demonstrate that EGFR inhibition and simultaneous TLR2 activation act synergistically to drive keratinocyte IL-36γ expression with subsequent production of the neutrophil chemoattractant IL-8 in the skin. Taken together with the observed high levels of expression of IL-36γ and the neutrophil-rich inflammation observed in the pilo-sebaceous units of inflamed skin resulting from EGFR inhibition, these results suggest a central pathogenic role of keratinocyte-derived IL-36γ in the acneiform skin toxicity caused by EGFRi.

### Increased expression and binding of the transcription factor KLF4 to the IL-36γ promoter upon EGFR inhibition.

**Figure 2:**
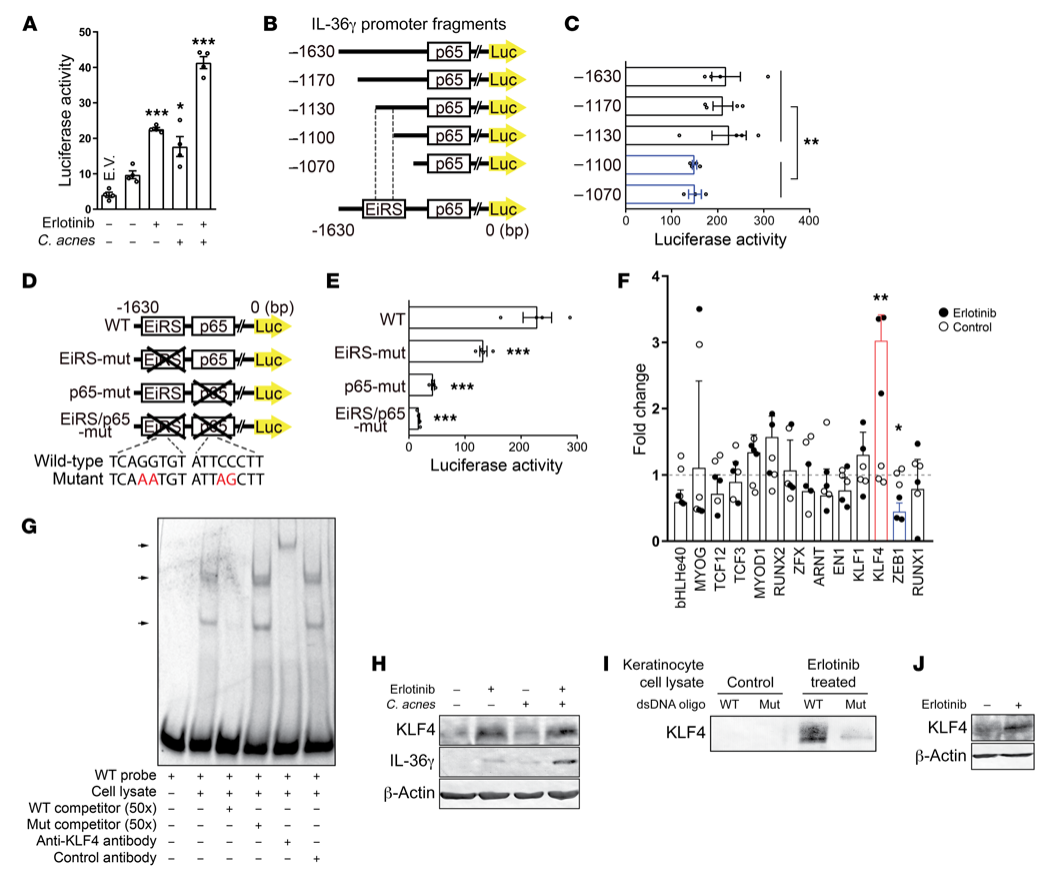
KLF4 binds to the IL-36γ promoter and regulates IL-36γ transcriptional activity in response to EGFR inhibition. (**A**) Luciferase reporter assay of human IL-36γ transcriptional activity in PHKs transfected with IL-36γ-pGL3 (1630 bp) reporter plasmid, followed by exposure to erlotinib and *C*. *acnes* for 16 hours. Renilla luciferase activity was measured to determine transfection efficiency. Data represent mean ± SEM (*n* = 4). E.V., empty vector. (**B** and **D**) Schematic of 5′-deletion and mutant constructs of the human IL-36γ promoter. Site-directed mutagenesis was performed to introduce the indicated mutation at the EGFR inhibitor–responsive site (EiRS) and p65 binding site. (**C** and **E**) 5′-Deletion and mutation study of the human IL-36γ promoter activity. PHKs were transfected with indicated plasmids, followed by exposure to erlotinib and *C*. *acnes* for 16 hours. Data represent mean ± SEM (*n* = 4). (**F**) Quantitative PCR was performed to evaluate the gene expression of transcription factor candidates binding to the EiRS. PHKs were exposed to erlotinib for 6 hours. Data represent mean ± SEM (*n* = 3). (**G**) Gel shift, competition, and supershift EMSA analysis using a Cy5-labeled oligonucleotide probe for the EiRS-containing region and HEK293T cell lysate containing KLF4 protein. (**H**) PHKs were exposed to erlotinib and *C*. *acnes* for 24 hours. (**I**) DNA pull-down assay using biotinylated wild-type– or mutant-oligonucleotide probe of the EiRS-containing region. These probes were incubated with extracts from PHKs exposed to erlotinib for 24 hours. DNA-associated proteins were visualized by Western blotting. (**J**) Ex vivo skin explants from healthy controls were exposed to erlotinib for 24 hours and KLF4 expression was assessed by Western blotting. The blot shown is representative of 2 different skin donors. Data were analyzed with 1-way ANOVA followed by Dunnett’s multiple-comparisons test (**A** and **E**) or with 2-tailed unpaired Mann-Whitney *U* (**C**) or *t* test (**F**). *P < 0.05; **P < 0.01; ***P < 0.001. All blots were run contemporaneously with the same protein samples.

To understand how EGFR inhibition and TLR2 signaling synergistically promote IL-36γ production in PHKs, we analyzed the transcriptional regulation of human IL-36γ. Histone modification patterns in PHKs revealed 1 enhancer and 1 promoter region upstream of the IL-36γ gene, with the promoter region containing a binding site for the NF-κB subunit p65 (ref. 36 and Supplemental Figure 2A). Interestingly, EGFR inhibition alone, or *C*. *acnes* exposure alone, resulted in only moderate enhancement of IL-36γ reporter activity as assessed in a luciferase reporter assay of human IL-36γ transcriptional activity in PHKs (Figure 2A). This is suggestive of the existence of 2 distinct responsive sites in the promoter region of the IL-36γ gene. In response to EGFR inhibition and *C*. *acnes* exposure, the activation pattern of a reporter containing both the IL-36γ enhancer and promoter regions was similar to the pattern observed when only the promoter was present (Supplemental Figure 2B), suggesting that the enhancer region is dispensable for erlotinib- and *C*. *acnes*–induced IL-36γ production in PHKs. Therefore, and given the synergistic effect of erlotinib and *C*. *acnes*, this observation suggests that the IL-36γ promoter contains a binding site for a transcription factor in addition to NF-κB p65. To identify this site, we generated IL-36γ reporters with promoter deletions of increasing lengths, thus mapping a genomic region located within 1130 and 1100 bp upstream of the first ATG as crucial for IL-36γ transcriptional activity (Figure 2, B and C). Furthermore, reporters containing mutations in either the genomic region located between 1130 and 1100 bp upstream of the first ATG or within the p65 binding site revealed, respectively, a 42% (*P* = 0.010) and 81% (*P* = 0.0003) reduction in IL-36γ transcriptional activity, whereas mutation of both regions resulted in a 92% (*P* = 0.0002) reduction (Figure 2, D and E), indicating that both regions are required for optimal IL-36γ transcriptional activity.

To identify the putative transcription factor that binds to the –1130- to –1100-bp region of the IL-36γ gene promoter, we searched the JASPAR database, an open-access repository for matrix-based transcription factor binding profiles (37), and identified 14 transcription factors as potential candidates (Supplemental Figure 2C). As no NF-κB–related transcription factors were revealed by this search, we hypothesized that the EGFRi-responsive site is located in this –1130- to –1100-bp region of the IL-36γ gene promoter. We subsequently performed quantitative PCR of mRNA derived from keratinocytes exposed to the EGFRi erlotinib, and among these 14 candidates thereby identified a significant change in the expression levels of 2 transcription factors, KLF4 and ZEB1 (Figure 2F). Because ZEB1 transcription decreased after EGFR inhibition, and is weakly expressed in normal keratinocytes (refs. 38, 39, and Supplemental Figure 2D), we considered KLF4 as the probable candidate and assessed whether KLF4 could effectively bind to the EGFRi-responsive region of the IL-36γ promoter. Using an electrophoretic mobility shift assay (EMSA), we could effectively demonstrate that KLF4 specifically binds to the DNA sequence within the –1130- to –1100-bp region of the IL-36γ gene promoter (Figure 2G). In line with the above results, EGFR inhibition resulted in increased KLF4 expression in PHKs (Figure 2H), and DNA pull-down assays performed with the same sequence as previously used in the EMSA revealed that KLF4 from erlotinib-exposed PHKs could specifically bind to the EGFRi-responsive region located between –1130 and –1100 bp upstream of the ATG in the IL-36γ gene promoter (Figure 2I). In addition, exposure of human skin ex vivo to erlotinib increased the expression of KLF4 (Figure 2J).

### Lack of a KLF4 binding site in the mouse IL-36γ promoter precludes murine EGFRi-induced IL-36γ response.

**Figure 3:**
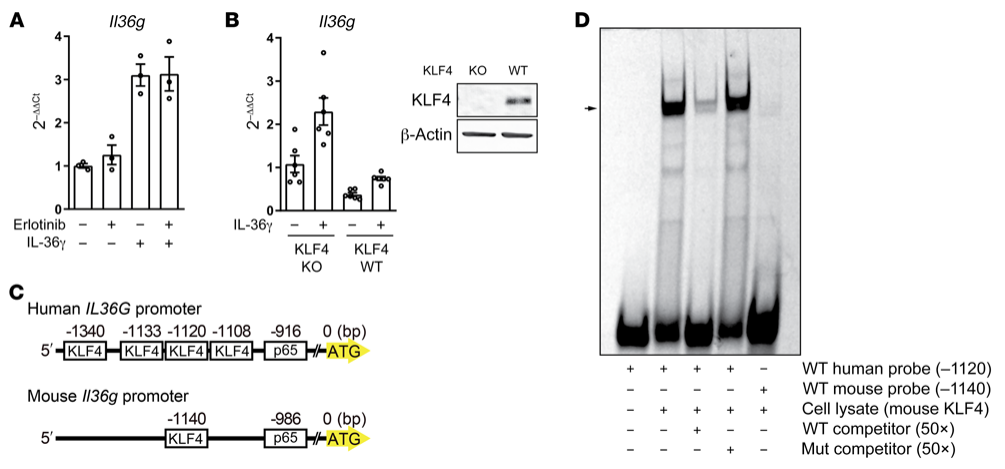
Lack of KLF4 binding site results in loss of synergistic IL-36γ production in mice. (**A**) PMKs were exposed to erlotinib (1 μM) and murine IL-36γ (100 ng/mL) for 6 hours; isolated RNA was analyzed by quantitative PCR. Data represent mean ± SEM (*n* = 3). (**B**) PMKs from wild-type or KLF4-knockout mouse were exposed to murine IL-36γ (100 ng/mL) for 6 hours. Data represent mean ± SEM (*n* = 3). PMK cell lysates were analyzed by SDS-PAGE and immunoblotting. Blots were run contemporaneously with the same protein samples. (**C**) Schematic of the human and murine IL-36γ promoter with predicted KLF4 binding site and p65 binding site by JASPAR. (**D**) Gel shift and competition EMSA analysis using a Cy5-labeled oligonucleotide probe for human and mouse KLF4 binding sites and HEK293T cell lysate containing murine KLF4 protein. Sequence-specific binding of human probe to murine KLF4 was demonstrated as a positive control. Gel shift reflecting formation of protein-DNA complexes with the murine probe, and KLF4 was not observed. Data are representative of 3 independent experiments.

Next, we examined IL-36γ production in response to EGFR inhibition and NF-κB activation in primary murine keratinocytes (PMKs). Surprisingly, despite the ability of the EGFRi erlotinib to block the phosphorylation of murine EGFR to a similar extent to that of human EGFR (Supplemental Figure 3A), enhanced IL-36γ production was not observed, in contrast to the effect observed in human keratinocytes (Figure 3A). In this setting, PMKs were exposed to murine IL-36γ to achieve NF-κB activation given their weak response to *C*. *acnes*, Pam3CSK4, and lipopolysaccharide (LPS) (data not shown). In our culture conditions, PMKs already expressed high levels of KLF4 in the basal state (Supplemental Figure 3B), a characteristic that was irrespective of the numerous culture conditions tested (data not shown). To test the requirement of KLF4 for IL-36γ transcription in mouse keratinocytes, we compared PMKs from KLF4-knockout mice and wild-type mice, and PMKs overexpressing KLF4; however, we were unable to detect synergistic IL-36γ elevation after NF-κB activation, as observed in human keratinocytes (Figure 3B and Supplemental Figure 3C). In accordance with the above, the putative KLF4 binding site identified by searching the JASPAR database, located 1140 bp upstream of the first ATG in the murine IL-36γ promoter (Figure 3C), could not be shown by EMSA to form a DNA-protein complex with mouse KLF4 (Figure 3D). Analysis of evolutionarily conserved regions in the genomes of sequenced species revealed that the KLF4 binding region in the human IL-36γ promoter is conserved in rhesus monkeys and chimpanzees, but not in mice or rats, whereas the sequence of the IL-36γ promoter region corresponding to the p65 binding site is approximately 70% conserved in mice and rats as compared with humans (Supplemental Figure 3D and refs. 40, 41). Alignment of the mouse and human IL-36γ gene loci revealed furthermore that the mouse genome lacks the region corresponding to the 583-bp-long region of human IL-36γ that contains the KLF4 binding site (–1120 bp) (Supplemental Figure 3E). These results demonstrate that the mouse IL-36γ promoter is devoid of the KLF4 binding site found in humans, explaining the absence of synergistic induction of IL-36γ expression by EGFR inhibition and NF-κB activation in murine keratinocytes, and suggests that the mouse is not an appropriate model for the in vivo analysis of the EGFRi-induced acneiform skin toxicity.

### Blockade of the EGFR/MEK/ERK pathway results in elevated KLF4 and IL-36γ expression.

**Figure 4:**
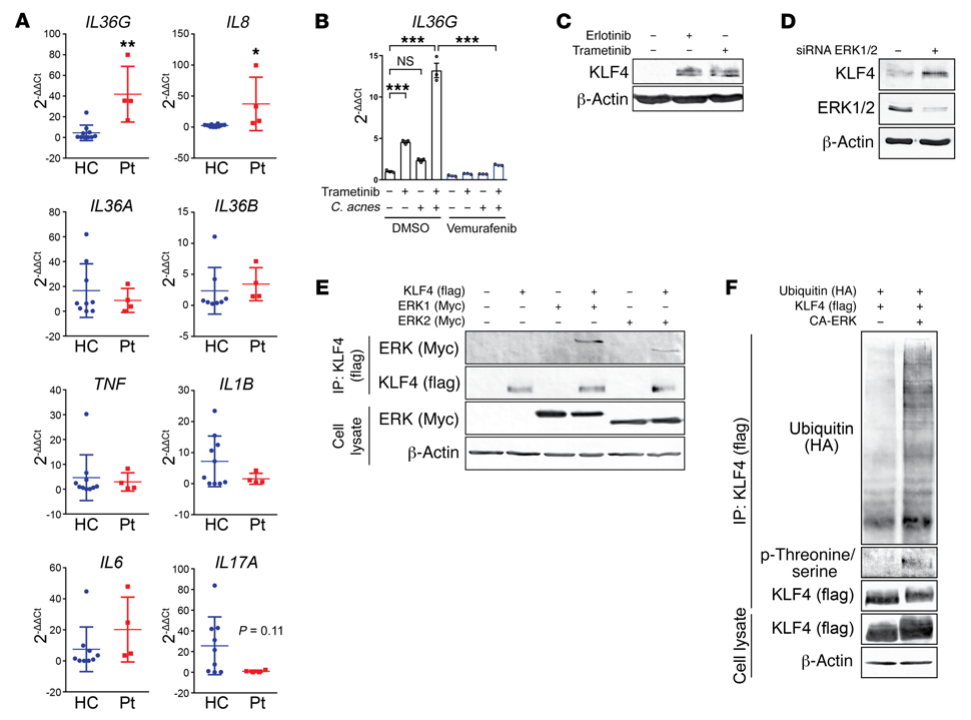
Blockade of the EGFR/MEK/ERK pathway increases keratinocyte expression of KLF4. (**A**) Quantitative PCR was performed to evaluate gene expression in RNA isolated from biopsies of 4 patients with acneiform eruption and 10 healthy control (HC) skin biopsies. Data represent mean ± SD. (**B**) PHKs were pre-exposed to the BRAF inhibitor vemurafenib (1 μg/mL) for 30 minutes and exposed to the MEK inhibitor trametinib (2 μg/mL) and *C*. *acnes* (MOI of 10) for 6 hours. Data represent mean ± SEM (*n* = 3). Data were analyzed with 2-tailed unpaired *t* test (**A**) or 1-way ANOVA followed by Tukey’s multiple-comparisons test (**B**). *P < 0.05; **P < 0.01; ***P < 0.001. (**C**) PHKs were exposed to erlotinib (1 μM) or trametinib (2 μg/mL) for 24 hours and total cell lysates were collected for Western blotting with antibodies against KLF4 and β-actin. (**D**) ERK1 and ERK2 were silenced by siRNA in PHKs and cell lysates were analyzed by SDS-PAGE and immunoblotting with indicated antibodies. (**E**) HEK293T cells were transfected with FLAG-tagged KLF4 and Myc-tagged ERK1 and ERK2 for 24 hours. Cell lysates were immunoprecipitated with an anti-FLAG antibody, followed by immunoblotting with the indicated antibodies. (**F**) HEK293T cells were transfected with FLAG-tagged KLF4, HA-tagged ubiquitin, and constitutively active ERK (CA-ERK) for 24 hours. Cell lysates were immunoprecipitated with an anti-FLAG antibody, followed by immunoblotting with the indicated antibodies. All blots were run contemporaneously with the same protein samples. Data are representative of 3 independent experiments.

MEKi, which block the MAPK/ERK signaling pathway by inhibiting the MAP kinases MEK1 and MEK2, cause adverse skin reactions similar to those observed in EGFRi-treated patients, including the commonly observed acneiform skin toxicity (4). Quantitative PCR analysis of acneiform lesional skin biopsies from MEKi-treated patients revealed, as observed in EGFRi-treated patients, elevated IL-36γ (9.4-fold, *P* = 0.0012) and IL-8 (15-fold, *P* = 0.019), but not IL-1β or IL-6 mRNA levels (Figure 4A and Supplemental Figure 4A). Because MEK is a downstream partner in the EGFR signaling pathway, we next assessed whether MEK inhibition could also result in elevated IL-36γ gene expression in PHKs. In vitro, the MEKi trametinib and selumetinib, together with *C*. *acnes*, synergistically induced elevated production of IL-36γ in PHKs, as previously observed with EGFRi (Figure 4B and Supplemental Figure 4B). Similar results were observed upon ERK silencing with siRNA (Supplemental Figure 4C). Of interest is the reported reduced incidence and severity of cutaneous skin toxicities observed in patients treated simultaneously with a BRAF inhibitor (BRAFi) and MEKi in clinical practice, as compared with patients treated with MEKi alone, and this has been shown to be due to paradoxical ERK activation in BRAF–wild-type cells (42–44). In line with this clinical observation, when PHKs were pre-exposed to the BRAFi vemurafenib prior to exposure to trametinib and *C*. *acnes*, the expression of IL-36γ mRNA induced by trametinib was significantly inhibited (7.4-fold, *P* = 0.0002) (Figure 4B and Supplemental Figure 4D).

Consistent with increased IL-36γ expression observed upon inhibition of the EGFR/MEK/ERK pathway at different levels, elevated KLF4 expression was also observed (Figure 4, C and D). Furthermore, ERK1 and ERK2 could be coimmunoprecipitated with KLF4 from HEK293T cells transfected with FLAG-tagged KLF4 and Myc-tagged ERK1 and ERK2 (Figure 4E), suggesting possible posttranscriptional modification of KLF4 by ERK1/2. Indeed, enhanced polyubiquitination and phosphorylation of proline-neighboring serine or threonine residues of KLF4 was observed in the presence of constitutively active ERK, and the latter is consistent with the activity of proline-directed protein kinase ERKs (ref. 45 and Figure 4F). To determine if KLF4 expression is regulated by ubiquitination and proteasomal degradation, expression in response to proteasome inhibition was analyzed. Indeed, increased KLF4 expression was observed upon proteasomal inhibition with MG132 (Supplemental Figure 4E), indicating that KLF4 expression is also controlled posttranslationally, and targeted for proteasomal degradation after ERK1/2 phosphorylation, as a downstream consequence of EGFR/MEK pathway activation. These data show that inhibition of either EGFR or MEK signaling in keratinocytes elevates KLF4 expression posttranslationally (Supplemental Figure 4F).

### KLF4 enhances IL-36γ transcriptional activity upon EGFR/MEK inhibition.

**Figure 5:**
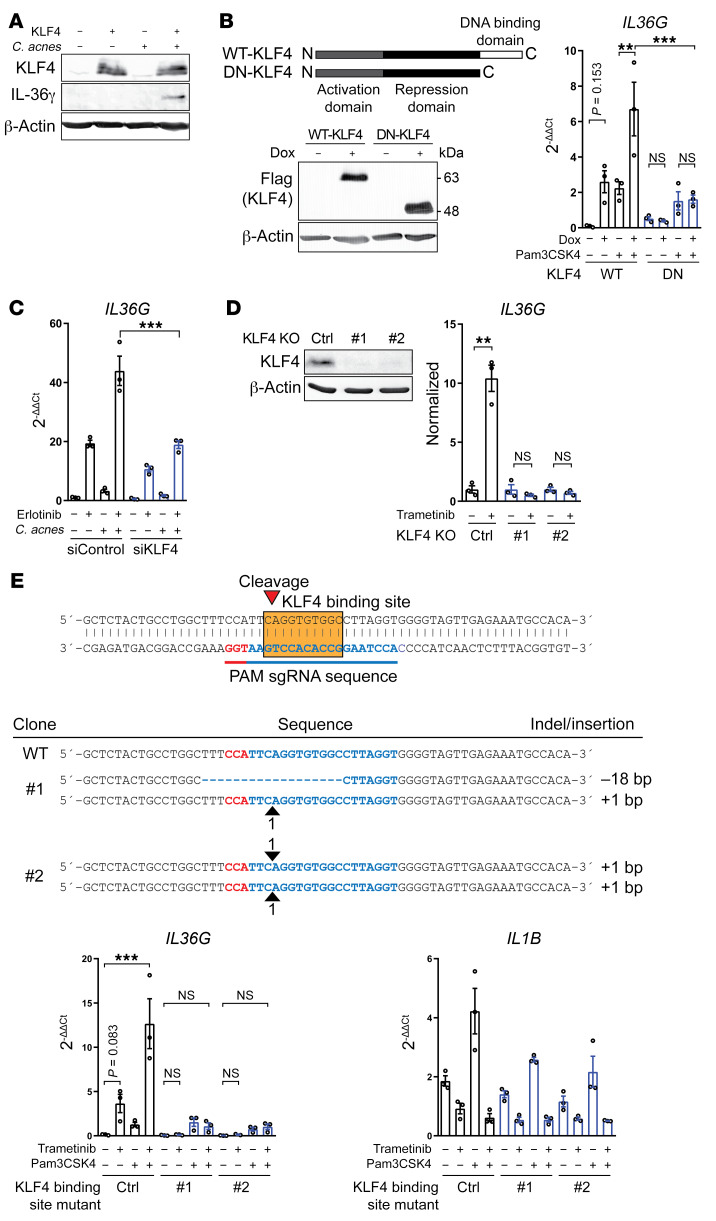
KLF4 is critical for IL-36γ transcriptional activity upon EGFR/MEK inhibition. (**A**) KLF4-overexpressing primary keratinocytes were exposed to *C*. *acnes* for 24 hours. (**B**) Flag-tagged wild-type (WT) and dominant-negative (DN) KLF4 were overexpressed in response to doxycycline using a Tet-on system for 24 hours, followed by exposure to Pam3CSK4 for another 24 hours. The cell lysates were collected for Western blotting and quantitative PCR (qPCR). Data represent mean ± SEM (*n* = 3). (**C**) KLF4 siRNA–treated PHKs were exposed to erlotinib and *C*. *acnes* for 6 hours and *IL36G* levels were analyzed by qPCR. Data represent mean ± SEM (*n* = 3). (**D**) Keratinocyte cell lines in which KLF4 was knocked out by CRISPR/Cas9 were exposed to trametinib (2 μg/mL) for 24 hours and total cell lysates were collected for Western blotting with antibodies against KLF4 and β-actin. The cells were exposed to trametinib for 24 hours and isolated RNA was analyzed by qPCR. Data represent mean ± SEM (*n* = 3). All blots were run contemporaneously with the same protein samples. Data are representative of 3 independent experiments. (**E**) Mutations generated by CRISPR/Cas9 in the KLF4 binding site. Red nucleotides are the PAM sequence and blue nucleotides hybridize to the sgRNA. KLF4 binding site–mutant cells were exposed to trametinib and Pam3CSK4 for 24 hours. Total RNA was analyzed by qPCR. Data represent mean ± SEM (*n* = 3). Data were analyzed with 1-way ANOVA followed by Dunnett’s (**B** and **E**) or Tukey’s multiple-comparisons test (**C**) or with 2-tailed unpaired *t* test (**D**). **P < 0.01; ***P < 0.001.

To determine if KLF4 is capable of enhancing IL-36γ transcriptional activity, we overexpressed KLF4 in PHKs. Such an overexpression resulted in enhanced IL-36γ expression at the protein level upon exposure of PHKs to *C*. *acnes* (Figure 5A). Similarly, doxycycline-inducible overexpression of wild-type KLF4 enhanced IL-36γ transcriptional activity, whereas a dominant-negative KLF4 mutant did not (Figure 5B). This demonstrates that forced expression of KLF4 can alone mimic the effect of EGFR/MEK inhibition to drive IL-36γ production in keratinocytes. In accordance with this, siRNA silencing of KLF4 substantially suppressed the ability of EGFRi and *C*. *acnes* to enhance IL-36γ production (Figure 5C). The deletion of KLF4 in keratinocyte cell lines using the CRISPR/Cas9 system resulted in a loss of induction of IL-36γ gene expression in response to MEKi (Figure 5D and Supplemental Figure 5). Furthermore, mutation of the KLF4 binding site in keratinocyte cell lines by CRISPR/Cas9 abrogated the ability of MEKi to induce IL-36γ transcription, whereas in these cell lines IL-1β expression was unaffected (Figure 5E). This demonstrates an essential role of KLF4 and its binding to the IL-36γ promoter in regulating IL-36γ transcriptional activity.

**Figure 6:**
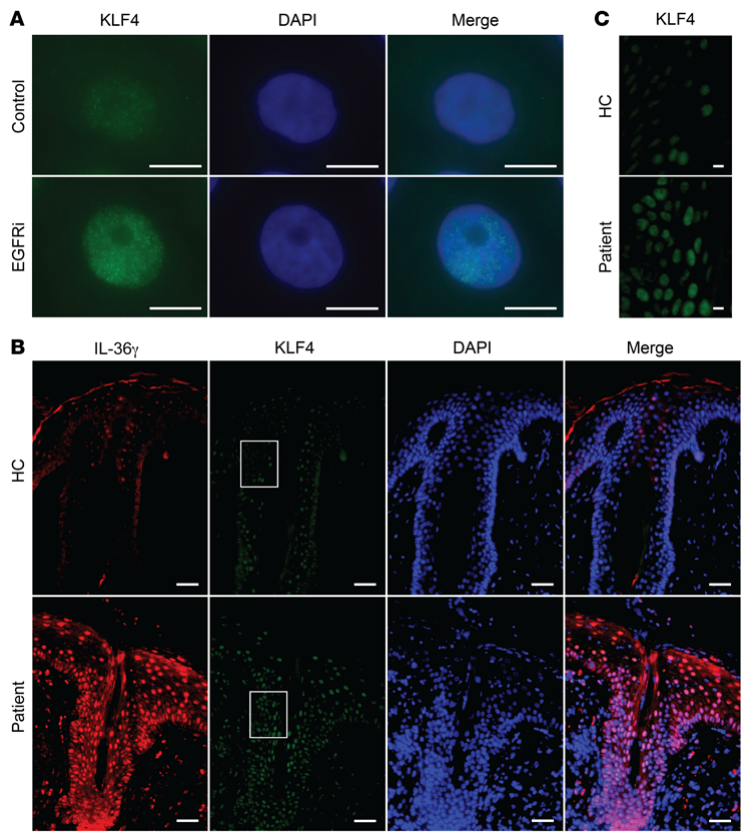
Increased KLF4 levels in vitro and in vivo upon EGFR inhibition. (**A**) Representative images of KLF4 expression (green) in PHKs after erlotinib or control DMSO exposure for 24 hours. Nuclei were stained with DAPI. Scale bars: 10 μm. Data are representative of 3 independent experiments. (**B** and **C**) Immunofluorescent staining with mouse anti-KLF4 (green) and rabbit anti–IL-36γ (red) antibodies of formalin-fixed, paraffin-embedded skin sections of acneiform eruption patients and healthy controls (HC). Nuclei were stained with DAPI. The white-boxed regions in **B** were zoomed separately in **C**. Scale bars: 50 μm (**B**) and 10 μm (**C**). Pictures are representative of 5 patients and 3 healthy individuals.

Consistent with a previous report (46), inhibition of the EGFR/MEK/ERK pathway resulted in increased KLF4 expression in the nuclei of PHKs in vitro (Figure 6A). In acneiform skin lesions from EGFRi-treated patients, abundant nuclear KLF4 expression could be observed in keratinocytes. In contrast, only low levels of nuclear KLF4 expression were observed in control skin samples (Figure 6, B and C). These data suggest that inhibition of either EGFR or MEK signaling enhances nuclear KLF4 expression in keratinocytes in the skin.

## Discussion

Here we demonstrate that EGFRi/MEKi partner with the commensal bacterium *C*. *acnes* that colonizes sebum-rich skin to potently induce keratinocyte IL-36γ expression and drive IL-8–mediated neutrophil-rich inflammation, the pathogenic hallmark of the so-called acneiform skin toxicity frequently associated with these targeted agents. On the basis of in vitro and ex vivo investigations we pinpoint the regulation of keratinocyte IL-36γ expression upon EGFR blockade to 2 important signaling events. First, an upregulation of the expression and subsequent binding of the transcription factor KLF4 to its binding site in the promoter region of IL-36γ, and second, a signal provided by *C*. *acnes* resulting in the binding of NF-κB p65 to a sequence in close proximity to the above-mentioned KLF binding site. Interestingly, the simultaneous binding of these 2 transcription factors to the IL-36γ promoter results not only in an additive but also a synergistic effect on IL-36γ gene transcription, which is of relevance for the characteristic localization of the EGFRi-induced rash to sebum-rich regions of the skin densely populated with pilo-sebaceous units and *C*. *acnes* such as the central face, upper chest, and back (17–22). Beyond the topographical distribution of the skin eruption, clinical practice guidelines for therapy support the involvement of *C*. *acnes* in the pathogenesis of acneiform skin toxicity induced by EGFR/MEK inhibition. Such guidelines recommend systemic treatment using tetracyclines such as doxycycline or retinoids such as acitretin (47), both of which exert an antibacterial effect on *C*. *acnes*, either directly for the former, or indirectly by reducing sebum production leading to an alteration of the follicular micromilieu and an indirect reduction in *C*. *acnes* counts by up to 3 logs (48).

As previously reported, the dysfunction of skin barrier and antimicrobial peptide production resulting from EGFR signaling abrogation are important events that can cause severe skin inflammation (13, 14). It is unclear, however, to what extent this may contribute to the initiation of acneiform skin toxicity by EGFR/MEK inhibition, possibly by facilitating the penetration of commensals such as *C*. *acnes* into the epidermis and/or pilo-sebaceous unit. Indeed, a possible involvement of other stimuli in addition to EGFR inhibition are suggested in skin rash development of a mouse model (49). KLF4 has been demonstrated to be a key driver of terminal epidermal differentiation in the skin (50). The enhanced differentiation induced by increased KLF4 in response to EGFR/MEK inhibition might be an important event leading to the skin barrier dysfunction. KLF4 has only rarely been mentioned to be related to inflammatory diseases, but is known as a regulator of proinflammatory cytokine expression in rheumatoid arthritis (51), and is one of the susceptibility genes for psoriasis (52, 53), 2 diseases in which IL-36γ is significantly increased in the inflamed tissues (54–57). Indeed, elevated expression of KLF4 has been reported in synovial tissue from rheumatoid arthritis patients and in the epidermis of psoriatic skin (51, 58), but the exact role of KLF4 in the pathogenesis of these inflammatory diseases remains to be defined.

IL-36γ has been demonstrated to form a self-amplifying inflammatory loop in keratinocytes that express high levels of the IL-36 receptor (34, 59). Besides DITRA, there is accumulating evidence that IL-36 signaling plays an important role in various neutrophilic dermatoses including generalized pustular psoriasis (GPP), palmo-plantar pustular psoriasis (PPP), acute generalized exanthematous pustulosis (AGEP), and acrodermatitis continua Hallopeau (60, 61). The data presented here provide substantial evidence that acneiform skin toxicity caused by EGFR/MEK inhibition should be added to the growing list of pustular skin diseases in which IL-36 likely plays a central pathogenic role. Our findings provide a basis for understanding the physiopathology of acneiform skin toxicity caused by EGFR/MEK inhibition that may lead to better benefit from the antitumor effects with reduced side effects. Several IL-36 inhibitors have been developed and phase II clinical trials of anti–IL-36 receptor antibody in patients with GPP and PPP are ongoing, suggesting that the latter may also offer a possibility for targeted therapy of acneiform skin toxicities caused by EGFR/MEK inhibition in the near future.

## Methods

### Human skin samples.

Biopsies were obtained from lesional skin of EGFRi/MEKi–treated patients with acneiform eruption. Normal skin was obtained from specimens from the Plastic Surgery Department, University of Zürich. All biopsies were immediately frozen in liquid nitrogen and stored at –80°C for RNA extraction or directly fixed in formalin (4% [w/v]) for at least 24 hours for histology.

### Mice.

*Klf4*-floxed mice were obtained from The Mutant Mouse Regional Resource Center (MMRRC) at the University of Missouri, Columbia, Missouri, USA. Rosa26-CreER^T2^ mice were obtained from The Jackson Laboratory. Tamoxifen-inducible *Klf4*-knockout mice were generated by crossing Rosa26-CreER^T2^ mice and *Klf4*-floxed mice. *Klf4* was knocked out by daily i.p. injection of tamoxifen at a dose of 100 mg/kg for 5 consecutive days. Wild-type C57BL/6 mice were obtained from Janvier Labs.

### Cell culture.

PHKs were cultured as previously described (62). Briefly, PHKs were isolated from fresh, surgically resected human neonatal foreskin. Keratinocytes were grown in keratinocyte serum-free medium (17005-042, Thermo Fisher Scientific), supplemented with EGF and bovine pituitary extract (BPE) (Thermo Fisher Scientific), and seeded for experiments after 3 passages. All cells were maintained at 37°C in a 5% CO_2_ humidified atmosphere. PMKs were isolated from pooled ears and tails. Briefly, skin specimens were incubated with the dermal side down at 37°C in Dulbecco’s modified Eagle medium (DMEM) supplemented with 1.25% trypsin (Sigma-Aldrich) and antibiotic-antimycotic solution (Gibco BRL) for 30 minutes. Separated epidermis was minced with sterile scissors and incubated at 37°C in DMEM supplemented with 10% (v/v) fetal bovine serum (FBS) and 0.25 mg/mL DNase I for 30 minutes, followed by filtration through a 70-μm cell strainer (BD Biosciences). Cells were resuspended in fresh keratinocyte serum-free medium (10744-019, Thermo Fisher Scientific) containing 50 ng/mL EGF (E4127, Sigma-Aldrich), 1 × 10^–10^ M cholera toxin (C8052, Sigma-Aldrich), and antibiotic-antimycotic solution (Gibco BRL) and seeded in dishes coated with collagen I (15 μg/cm^2^, 354236, Corning). After 1 day of attachment, nonadherent cells were washed away and fresh medium was added. HEK293T cells were cultured in DMEM supplemented with 10% (v/v) FBS, antibiotic-antimycotic solution (Gibco BRL), sodium pyruvate (Invitrogen), and GlutaMAX solution (Invitrogen). Puromycin (catalog P9620) and blasticidin (catalog 15205) were from Sigma-Aldrich.

### Plasmids.

Human genomic DNA was isolated from PHKs by QIAamp DNA Mini Kit (QIAGEN). The 6782-bp sequence upstream from the first ATG of the human IL-36γ gene was amplified using Pfu polymerase (Invitrogen) with primers (forward, 5′-CACCTGGGCATATTGCATAATGG-3′; reverse, 5′-AAGCTTAGTGTGGTTGTCTCAGCAC-3′, excluding an additional flanking BglII-HindIII site) and subcloned into the luciferase reporter vector pGL3-Basic (Promega). The human IL-36γ promoter (1630 bp) luciferase construct and its NF-κB mutant construct were gifts from Heiko Mühl (Goethe University Frankfurt, Germany). Site-directed mutagenesis was performed using Pfu Turbo (Thermo Fisher Scientific) according to the manufacturer’s instructions to generate a point mutation in the EGFRi–responsive site. Sequentially shorter reporter constructs of the human IL-36γ promoter were generated from the human IL-36γ promoter (1630 bp) construct, using the following forward primers excluding an additional flanking BglII site: 5′-CCATGTGGATGGAGCTGAAA-3′ (1180 bp); 5′-GCCTGGCTTTCCATTCAGGT-3′ (1135 bp); 5′-GTGGGGTAGTTGAGAAATGC-3′ (1105 bp); and 5′-CTTGCCTGAGACGTGTGGCT-3′ (1076 bp). The dominant-negative human KLF4 construct was generated from a human KLF4 construct (Addgene, 26815), using the following reverse primer and excluding additional restriction enzyme sites: 5′-AAAGAGGGGAAGACGATCGTAA-3′. The following cDNAs were subcloned into pcDNA3.1 (Invitrogen) or pMXs-IP (gift from Toshio Kitamura, University of Tokyo, Japan): mouse Klf4 (Addgene, 15920), human ERK1 (Addgene, 23509), human ERK2 (Addgene, 23498), and human ubiquitin (Addgene, 31815). The constitutively active (CA)-ERK plasmid was a gift from Jukka Westermarck (University of Turku, Finland).

### Reagents.

Erlotinib was purchased from MedChem Express. Cetuximab was from MERCK Serono. Trametinib and vemurafenib were from ApexBio. Selumetinib and MG132 were from Selleckchem. Recombinant human IL-36γ (catalog 6835), mouse IL-36γ (catalog 6996), and human IL-36Ra (catalog 1275) were from R&D Systems. Pam3CSK4 was from InvivoGen. The goat anti–IL-36γ (catalog AF2320) and anti-mouse KLF4 (catalog AF3158) antibodies were from R&D Systems. The rabbit anti–IL-36γ (catalog LS‑C201142) and its blocking peptide (catalog LS-E45854) were from LifeSpan BioSciences. The anti–human KLF4 antibody (catalog AM09057PU-N) was from Acris. The anti–β-actin (catalog A5441), anti-FLAG (catalog F1804), and anti-myc (catalog C3956) antibodies were from Sigma-Aldrich. The anti–human KLF4 (catalog 12173), anti-ERK (catalog 9107), anti–phospho-ERK (catalog 4370), and anti–phospho-threonine/proline (catalog 9391) antibodies were from Cell Signaling Technology. The anti-T7 antibody was from Abcam (catalog ab9138). The anti-HA antibody was from Santa Cruz Biotechnology (catalog sc-805). The secondary antibodies used were alkaline phosphatase–conjugated mouse IgG (catalog S372B), rabbit IgG (catalog S373B), and goat IgG (V115A) from Promega. Live *C*. *acnes* was prepared as previously described (63).

### Gene expression array.

Total RNA was extracted from individual skin samples using TRI Reagent (Sigma-Aldrich) according to the manufacturer’s instructions. One microgram of each RNA sample was converted to cDNA with an RT^2^ First Strand kit (Qiagen) and used in real-time PCR performed on a Human Inflammatory Response & Autoimmunity RT^2^ Profiler PCR Array (PAHS-3803Z, Qiagen) according to the manufacturer’s protocol. Data analysis was performed using the ΔΔCt method.

### Quantitative PCR.

cDNA was generated from total RNA using a RevertAid First-Strand cDNA Synthesis Kit (Thermo Fisher Scientific) according to the manufacturer’s instructions. Quantitative real-time PCR was performed using a LightCycler 480 (Roche) with SYBR Green I Master Mix (Roche). The primers used for amplification of specific genes were synthesized by Microsynth (Supplemental Table 2).

### In situ hybridization.

Human IL-36γ cDNA (620 nt) was amplified using Pfu polymerase (Invitrogen) with primers (forward, 5′-GGAAGCTGCTGGAGCCACGATTC-3′; reverse, 5′-AAAGACCAAGCTGCCACCTCTAGG-3′, excluding an additional flanking HindIII-EcoRI site) and subcloned into the pcDNA3 vector (Invitrogen). PCR fragments for probes were amplified with primers for CMV and BGH. The digoxigenin-labeled (DIG-labeled) antisense and sense RNA probes for human IL-36γ were synthesized by in vitro transcription using either SP6 or T7 RNA polymerase with the DIG RNA labeling kit (Roche). These probes were hydrolyzed in hydrolysis buffer (40 mM NaHCO_3_, 60 mM Na_2_CO_3_) to produce 0.25-kb fragments. The unincorporated nucleotides were removed using a spin column (Roche). Formalin-fixed, paraffin-embedded tissue sections (4 μm) were deparaffinized and rehydrated in RNase-free conditions. Sections were treated with 1 μg/mL proteinase K for 20 minutes and washed with 2× SSC 3 times, followed by prehybridization for 2 hours in 2× SSC containing 50% formamide. Hybridization buffer (HB) contained 50% formamide, 4× SSC, 100 ng/mL yeast tRNA, and 10% dextran sulfate. Twenty-five nanograms of DIG-labeled RNA probes was diluted in 50 μL HB, heated to 95°C for 5 minutes, added to the tissues, and hybridized overnight. After hybridization, the tissues were washed and incubated in stringent wash buffer (20% formamide, 2× SSC) at 42°C for 30 minutes, followed by 2 μg/mL RNase A treatment at 37°C for 1 hour. The sections were washed in 2× SSC and 0.2× SSC at 55°C each for 30 minutes and PBS at room temperature for 5 minutes. Blocking was performed in 5% BSA in PBS at room temperature for 1 hour and sections were incubated in alkaline phosphatase–conjugated anti-DIG Fab fragment (1:4000 dilution; Roche) at room temperature for 2 hours. After 3 washes in PBS for 5 minutes each, tissues were stained using 2% NBT/BCIP in 0.1 M NaCl, 0.1 M Tris-HCl (pH 9) at room temperature in the dark for 2 days.

### Immunohistochemistry.

Five-micrometer formalin-fixed, paraffin-embedded human skin sections were deparaffinized and rehydrated. Antigen unmasking was performed by heating the slides for 25 minutes in Target Retrieval solution (DAKO). Sections were blocked using 5% BSA in PBS for 1 hour and stained for 2 hours at room temperature with anti–IL-36γ antibody. Primary antibodies were detected using a biotin-conjugated secondary antibody (Southern Biotech) followed by an avidin-biotin complex and addition of peroxidase substrate (Vector Laboratories). Nuclei were counterstained using a solution of hematoxylin. The sections were mounted in mounting medium (DAKO) and imaged using an Aperio ScanScope (Leica Biosystems).

### Immunofluorescent staining.

PHKs were seeded on circular 18-mm glass coverslips (Hecht-Assistent). Cells were fixed for 30 minutes in 3% paraformaldehyde/2% sucrose solution, permeabilized for 2 minutes with 0.2% Triton X-100 in PBS, and blocked for 1 hour in 1% BSA (BSA Fraction V; GE Healthcare) in 0.5% Tween 20 in PBS. In vitro keratinocyte samples were stained for 2 hours at room temperature with anti-KLF4 goat antibody followed by DyLight 488–conjugated secondary antibody (ab96891, Abcam). Skin tissue samples were prepared as described in the *Immunohistochemistry* section above. The sections were stained for 2 hours at room temperature with anti-KLF4 mouse antibody and anti–IL-36γ rabbit antibody followed by Alexa Fluor 488/555–conjugated secondary antibodies (A-11001 and A-21429, Thermo Fisher Scientific). Nuclei were counterstained with DAPI. The sections were mounted in the mounting medium and imaged using an Aperio ScanScope.

### Western blot.

To prepare whole-cell lysates, cells were lysed in SDS buffer with DTT (Sigma-Aldrich). Proteins were resolved in SDS-PAGE gels with a Mini-PROTEAN Tetra Vertical Electrophoresis Cell (Bio-Rad Laboratories, Inc.) at a constant voltage (80–120 V) and transferred to an Amersham Protran 0.2-μm nitrocellulose membrane (GE Healthcare) using semidry or wet systems from Bio-Rad. The membranes were blocked with 5% dried milk in PBS supplemented with 0.5% Tween 20 (Sigma-Aldrich) and then probed overnight with primary antibodies at 4°C followed by alkaline phosphatase–conjugated secondary antibodies for 1 hour at room temperature. Proteins were detected using BCIP/NBT color development substrate (Promega) and dried membranes were scanned using a LiDE 210 scanner (Canon Inc.).

### ChIP-seq/DNase-seq/RNA-seq data analysis.

Raw sequencing data were converted to Fastq files by the NCBI SRA Toolkit. Quality control on the raw data was performed by FastQC. The reads for ChIP-seq/DNase-seq were aligned to human reference genome (build GRCh37/hg19) by Bowtie2 Aligner. The mapped sequence reads were transformed to a binary format, sorted, and indexed by SAMtools, followed by generation of coverage plots by BEDtools. These files were converted into BigWig files by BedGraphtoBigWig and visualized in IGV. ChIP-seq data from PHKs were obtained from the following ChiP-seq/DNase-seq samples: GSM941735, GSM733698, GSM733674, GSM733636, and GSM816635. ChIP-seq data for p65 was from GSM935526. The reads for RNA-seq were aligned to human reference genome (build GRCh37/hg19) by HISAT2. The transcripts were assembled by Cufflinks, followed by generation of differential gene expression data by Cuffdiff. RNA-seq data from PHKs were obtained from GSM2074746, GSM2074747, and GSM2074748.

### Luciferase reporter assay.

PHKs and PMKs were transfected with reporter constructs using a TransIT-X2 Dynamic Delivery System (Mirus Bio) according to the manufacturer’s instructions. Cotransfection of the Renilla luciferase expression vector pRL-TK (Promega) was used as an internal control for all reporter assays. Cell extracts were generated 24 hours after transfection using Reporter Lysis Buffer (Promega) and extracts were assayed for firefly luciferase and Renilla luciferase activity using the Luciferase Assay system (Promega) and coelenterazine (0.1 μg/mL, Sigma-Aldrich), respectively. Luminescence was measured with the Cytation3 Imaging Reader (BioTek).

### Gene transfer and knockdown.

HEK293T cells were transfected with 8 μg of mammalian expression constructs for human and mouse KLF4 using a TransIT-X2 Dynamic Delivery System (Mirus Bio). PHKs were transduced using a published protocol (64) with minor modifications. Briefly, viral supernatant was produced by transfecting 8 μg of KLF4-pMXs-IP into Phoenix Ampho cells (ATCC) using the TransIT-X2 Dynamic Delivery System. Viral supernatant was collected 48 hours after transfection and added to PHKs in 6-well plates supplemented with 10 μg/mL polybrene (Sigma-Aldrich) followed by centrifugation at 650 *g* for 45 minutes at 32°C. After centrifugation, PHKs were washed with PBS and cultured in fresh medium. The same transfection step was repeated on the next day and PHKs were incubated another 24 hours for experiments. PMKs were transfected in 12-well plates with 3 μg of mammalian expression construct of mouse KLF4 using the TransIT-X2 Dynamic Delivery System. Silencing RNA (siRNA) transfection of PHKs was carried out using INTERFERin (Polyplus-transfection) at a final concentration of 5 nM endoribonuclease-prepared siRNA (esiRNA) according to the manufacturer’s protocol. The esiRNAs used (EGFR, KLF4, ERK1, and ERK2) were purchased from Sigma-Aldrich. Experiments were performed 2 days after transfection. EGFP esiRNA was used as a control. Short hairpin RNA (shRNA) fragments of human TLR2 were hybridized with synthesized sense and antisense oligonucleotides. The sense strand sequence is 5′-CCGGCCAGCCAGAAAGCACTACAATCTCGAGATTGTAGTGCTTTCTGGCTGGTTTTT-3′. DNA oligonucleotides were synthesized by Microsynth and ligated into Tet-pLKO-puro (Addgene, 21915). Viral supernatant was produced by transfecting the Tet-pLKO-puro plasmid, psPAX2 (Addgene, 12260), and pMD2.G (Addgene, 12259) into HEK293T cells and viruses were harvested 48 hours later. The KERTr keratinocyte cell line (ATCC CRL-1658) was transduced with the virus and selected for 1 week with 1 μg/mL puromycin. Gene knockdown was induced by culturing the cells in medium containing 1 μg/mL doxycycline.

### Generation of CRISPR/Cas9 cell lines.

Single-stranded DNAs (Supplemental Table 3) were subcloned into the pLentiCRISPRv2 plasmid (Addgene, 98293). Viral supernatant was produced by transfecting the pLentiCRISPRv2 plasmid, psPAX2, and pMD2.G into HEK293T cells and viruses were harvested 48 hours later. KERTr cells were transduced with the virus and selected for 10 days with 10 μg/mL blasticidin. Cloning was performed by limiting dilution in conditioned medium. Genomic DNA was extracted from the isolated single-cell-expanded clones using a QIAamp DNA Mini Kit and amplicons harboring the targeted alleles were prepared by PCR using Taq polymerase (EP0404, Thermo Scientific). The PCR amplicons were subcloned into a TOPO vector using a TA Cloning Kit (Promega) according to the manufacturer’s protocol and then submitted for Sanger sequencing.

### EMSA.

The sequences of the probes used for EMSA were the following: EGFRi-responsive site wild-type forward, 5′-TTCCATTCAggTGTGGCCTTAG-3′; wild-type reverse, 5′-CTAAGGCCACAccTGAATGGAA-3′; mutant forward, 5′-TTCCATTCAaaTGTGGCCTTAG-3′; mutant reverse, 5′-CTAAGGCCACAttTGAATGGAA-3′; putative murine KLF4 binding site forward, 5′-GAGATCCAGGTGGAAAGGAAGA-3′; and reverse, 5′-TCTTCCTTTCCACCTGGATCTC-3′. The probes including Cy3 modification at the 5′ end were synthesized by Microsynth. To construct oligonucleotide duplexes, 2 nmol of each sense and antisense oligonucleotides were annealed in a buffer (100 mM NaCl, 50 mM Tris-HCl, 10 mM MgCl_2_, and 100 μg/mL BSA) by heating the mixtures to 95°C for 5 minutes and allowing the solution to cool slowly to room temperature. EMSA was performed with 5 μg of cell lysate, 0.15 pmol of Cy3-labeled oligonucleotides, 1.5 μg of BSA, 0.5 μg of poly(dI-dC), and in 12 μL of reaction mixture (24 mM HEPES-KOH pH 7.9, 8 mM Tris-HCl [pH 8.0], 2 mM EDTA, 1 mM DTT, and 12% glycerol) with a proteinase inhibitor cocktail (Roche). Competition assays were performed to demonstrate the sequence-specific binding of the probes. For the competition assays, a 50-fold molar excess of unlabeled wild-type or mutant oligonucleotide probe was added 20 minutes before the addition of Cy3-labeled probes and incubated for another 20 minutes at room temperature. Supershift assays were performed to demonstrate the complex formation of the protein of interest and the target probe, by means of appearance of a new supershifted band upon addition of antibody targeting the protein of interest. For the supershift assays, 1 μg of anti-KLF4 antibody (Cell Signaling Technology) was added to the reaction mixture for 20 minutes before the addition of Cy3-labeled probes. Samples were loaded onto Novex 6% DNA retardation gels (Thermo Fisher Scientific) and electrophoresed in 0.5× Tris-borate buffer for 30 minutes at 150 mV. Gels were subsequently visualized on an Odyssey Fc Imaging System (LI-COR Biosciences).

### DNA pull-down assay.

The sequences of the probes used for the DNA pull-down assay were the same as those for EMSA. 5′-Biotinylated wild-type and mutant forward oligonucleotides were synthesized by Microsynth and annealed with nonbiotinylated reverse oligonucleotides. Cell lysates were incubated in the same reaction buffer as EMSA with 7.5 nmol of double-stranded annealed oligonucleotides at 4°C overnight with gentle shaking on a rocker. Thirty microliters of Pierce High Capacity Streptavidin Agarose beads (Thermo Fisher Scientific) was added and incubated at 4°C for 1 hour. Beads were washed 5 times with ice-cold PBS using SigmaPrep spin columns (Sigma-Aldrich). Proteins bound to streptavidin beads were dissolved in 2× SDS sample buffer, boiled at 95°C for 5 minutes, and immunoblotted.

### Coimmunoprecipitation analysis.

HEK293T cells were transfected in 6-well plates with 2.4 μg of plasmids (0.4 μg Myc-tagged ERK1, 1.6 μg ERK2, and 0.4 μg FLAG-tagged KLF4; or 0.8 μg HA-tagged ubiquitin, 0.8 μg FLAG-tagged KLF4, and 0.8 μg CA-ERK) using TransIT-X2 Dynamic Delivery System (Mirus Bio) according to the manufacturer’s instructions. As controls, 2.4 μg of empty vector pcDNA3.1 was added to cells. Twenty-four hours after transfection, cells were washed with ice-cold PBS and lysed with prechilled lysis buffer (50 mM Tris-HCl [pH 7.5], 100 mM NaCl, and 0.1% [v/v] Triton X-100 supplemented with protease and phosphatase inhibitor cocktail [Roche]) for 30 minutes on a rocker at 4°C. Samples for ubiquitin analysis were boiled at 95°C for 5 minutes in 1% SDS and sample buffer was added to dilute the SDS to 0.1%. Samples were incubated overnight with anti-FLAG (1 μg) at 4°C, followed by the addition of 30 μL Protein A/G PLUS Agarose beads (Santa Cruz Biotechnology) and incubation for 2 hours. Immunoprecipitates were collected by centrifugation at 1000 *g* for 5 minutes at 4°C and the beads were then washed 5 times with lysis buffer. The beads were resuspended in 2× SDS sample buffer, boiled at 95°C for 5 minutes, and immunoblotted.

### ELISA.

PHKs were exposed to 1 μM erlotinib and 5 μg/mL Pam3CSK4 in 6-well plates for 48 hours. After washing 3 times with prewarmed PBS, cells were incubated in fresh medium for 48 hours. After centrifugation, supernatants were collected and analyzed by IL-36γ ELISA (Adipogen).

### Ex vivo skin culture.

Ex vivo skin culture was performed using a published protocol (65) with the following modifications. Full-thickness skin specimens were obtained from patients undergoing plastic or reconstructive surgery. Skin samples were cut into small pieces (4 × 4 mm) and placed in 24-well plates containing 0.5 mL of keratinocyte serum-free medium (17005-042, Thermo Fisher Scientific) supplemented with EGF, BPE (Thermo Fisher Scientific), 100 μg/mL kanamycin (Invitrogen), and 1.4 mM CaCl_2_ (Sigma-Aldrich). Tissue cultures were then incubated at 37°C in a 5% CO_2_ atmosphere with fresh culture medium provided at 2-day intervals. Ex vivo skin explants were cultured with 1 μg/mL erlotinib for 24 hours for Western blotting or for 4 days followed by snap freezing for quantitative PCR.

### Statistical analysis.

Statistical analysis was performed using unpaired Student’s *t* test or 1-way analysis of variance (ANOVA) followed by Dunnett’s multiple-comparisons test, using Prism 7.02 software (GraphPad). Differences were considered significant when *P* was less than 0.05: *P < 0.05; **P < 0.01; ***P < 0.001.

### Study approval.

All experiments with human samples and the use of human skin samples for research studies were carried out in accordance with the Cantonal Ethical Committee of Zurich, Switzerland after informed written patient consent and according to the Declaration of Helsinki Principles. All animal procedures were approved by the Cantonal Veterinary Office of Zurich, Switzerland.

## Author contributions

TKS, EC, and LEF designed and supervised the research. TKS, MM, GF, and AO conducted the studies. BMS, HDB, TR, JTM, JH, and AAN collected human biological samples and patients’ data. TKS, MM, EC, and LEF analyzed the data and prepared and revised the manuscript. All authors reviewed the manuscript.

## Supplementary Material

Supplemental data

## Version 1 12/05/2019

In-Press Preview

## Version 2 02/04/2020

Electronic publication

## Version 3 03/02/2020

Print issue publication
