# Supplementary material for: IL-36γ drives skin toxicity induced by EGFR/MEK inhibition and commensal Cutibacterium acnes
Source: J Clin Invest. 2020 Feb 4;130(3):1417–30. doi: 10.1172/JCI128678 (PMC7269569; doi:10.1172/JCI128678)
Supplement: Supplemental data [file jci-130-128678-s179.pdf]

Supplemental Figure 1

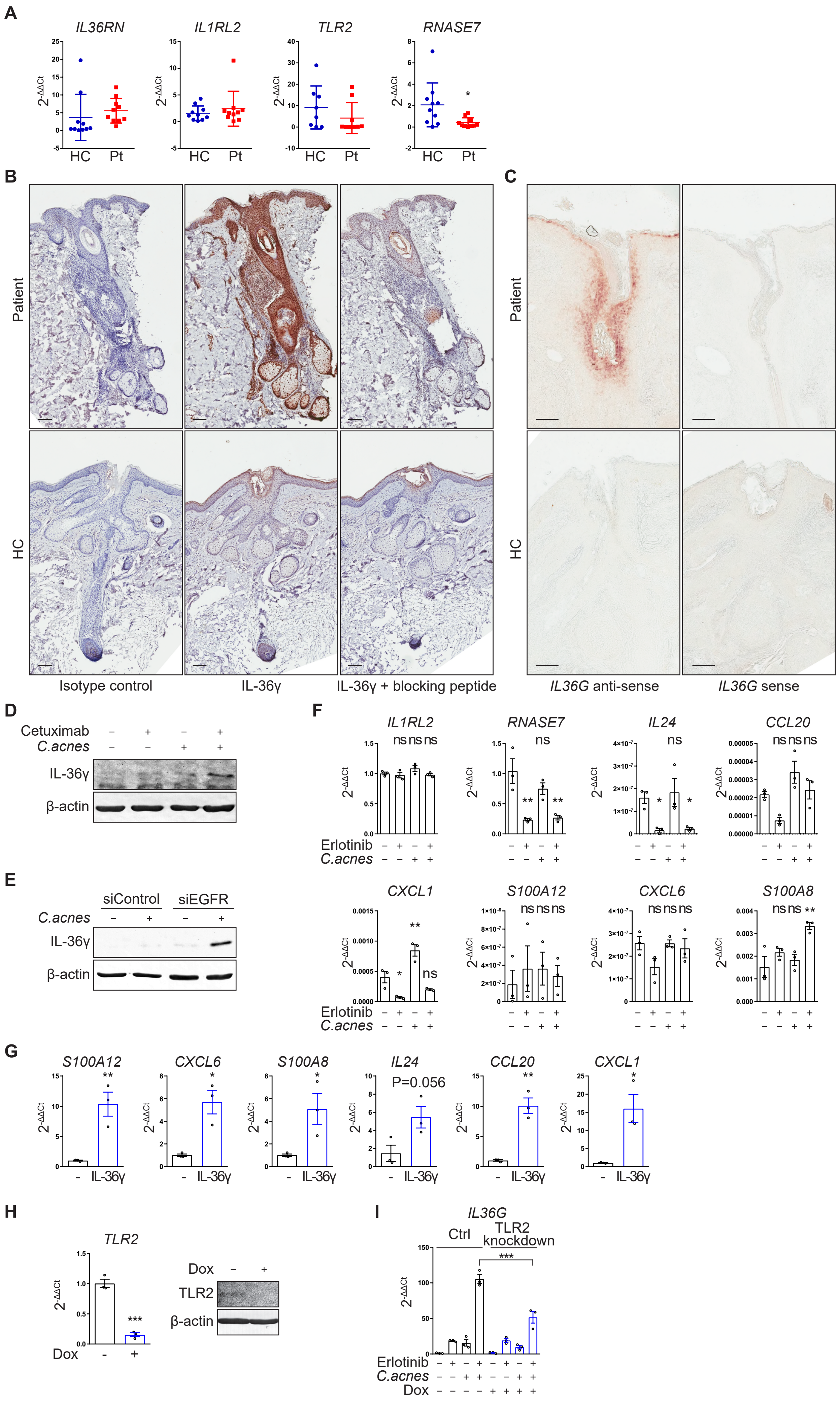

Supplemental Figure 1. Increased production of IL-36γ in primary keratinocytes and lesional skin of patients in response to EGFR inhibition and *C. acnes*. (**A**) RNA from lesional skin biopsies of patients suffering from acneiform eruption and healthy control skin biopsies were subject to qPCR analysis (n=10 per group). (**B**) Immunohistochemistry staining of formalin-fixed paraffin-embedded lesional skin sections of acneiform eruption patients and normal donors with rabbit anti-IL-36γ antibody accompanied with a preincubation test with an IL-36γ competing peptide. Scale bar represents 100 μm. Pictures are representative of five patients and three healthy individuals. (**C**) IL-36γ mRNA was detected by in situ hybridization. The sense strand is a negative control. Scale bar represents 100 μm. Pictures are representative of four patients and three healthy individuals. (**D**) Cell lysates from PHKs exposed to cetuximab (anti-EGFR antibody, 5 μg/mL) and *C. acnes* (MOI of 10) for 24 hours were subject to western blotting using antibodies against IL-36γ and β-actin. (**E**) EGFR siRNA-transduced PHKs were exposed to *C. acnes* for 24 hours and cell lysates were subjected to immunoblotting. (**F**) Total RNA was prepared from PHKs exposed to erlotinib (1 μM) and *C. acnes* (MOI of 10) for 6 hours and subjected to qPCR. n=3. (**G**) Total RNA was prepared for qPCR from PHKs exposed to IL-36γ (100 ng/mL) for 6 hours. n=3. (**H**) Total RNA and cell lysates were prepared for qPCR and immunoblotting from the human keratinocyte cell line KERTr cells expressing TLR2-shRNA after doxycycline treatment for 5 days. (**I**) Total RNA was prepared from TLR2-knocked down cells by the expression of TLR2-shRNA followed by exposure to erltobinib and *C. acnes* for 24 hours. n=3. Data represent means ± SEM. Data were analyzed with 2-tailed unpaired t test (**A**, **G** and **H**), 1-way ANOVA followed by Dunnett's (**F**) or Tukey's multiple-comparisons test (**I**). *P<0.05, **P<0.01, ***P<0.001. All blots were run contemporaneously with the same protein samples.

Supplemental Figure 2

A

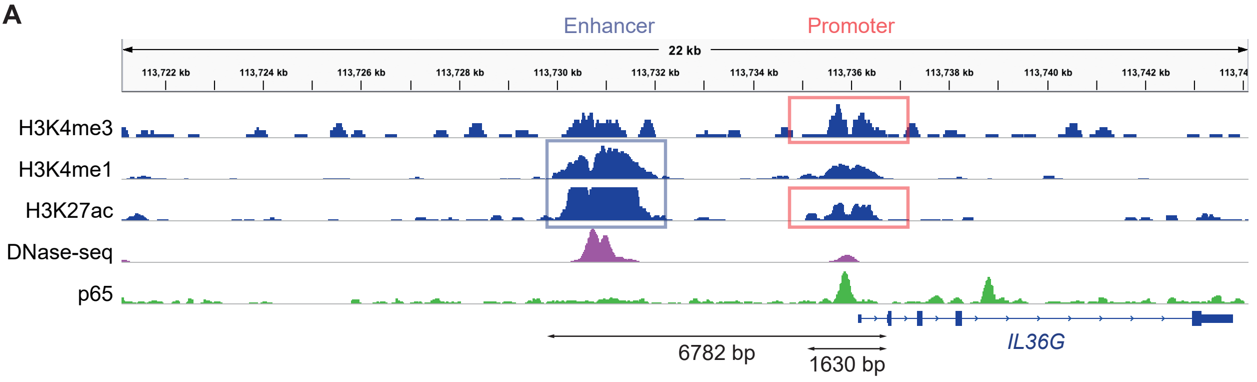

B

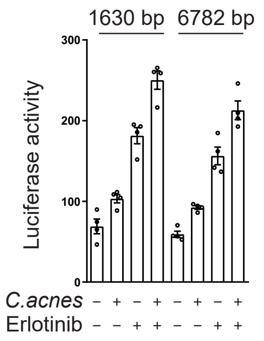

C

Sequence of the region
GCCTGGCTTTCCATTCAGGTGTGGCCTTAGGTGGGGTA

Transcription factor	Sequence	Score
bHLHe40	ATTCAGGTGTG	9.771
MYOG	ATTCAGGTGTG	1.777
TCF12	ATTCAGGTGTG	2.246
TCF3	ATTCAGGTGTG	5.313
MYOD1	TTCAGGTGTGGCC	2.722
RUNX2	TTCAGGTGTGGCCTT	7.006
ZFX	TCAGGTGTGGCCTT	7.464
ARNT	CAGGTG	6.112
EN1	CAGGTGTGGCC	5.967
KLF5	CAGGTGTGGC	1.681
KLF1	CAGGTGTGGCC	6.984
KLF4	CAGGTGTGGC	5.876
ZEB1	CAGGTGTGG	14.587
RUNX1	AGGTGTGGCCT	7.484

D

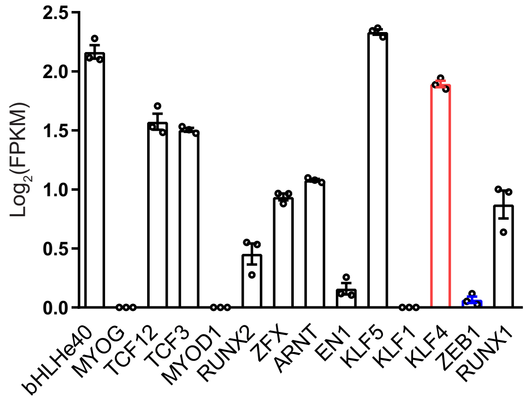

Supplemental Figure 2. KLF4 binds to the human IL-36γ promoter and regulates IL-36γ transcriptional activity in response to EGFR inhibition. (**A**) Chromatin immunoprecipitation-high throughput sequencing (ChIP-seq) tracks for H3K4me3, H3K4me1 and H3K27Ac at the human IL-36γ gene are shown. DNase-seq shows accessible DNA regions in the genome. (**B**) Luciferase reporter assay of human IL-36γ transcriptional activity in PHKs transfected with human IL-36γ-pGL3 (1630 bp) or (6782 bp), followed by exposure to erlotinib (1 μM) and *C. acnes* for 16 hours. TK Renilla luciferase was measured to determine transfection efficiency. Data represent means ± SEM. n = 4. (**C**) The transcription factors predicted by JASPAR to bind to the sequence of wild-type but not mutant sequence of the EGFR inhibitor-responsive region. Two guanines in the middle are substituted by two adenines in the mutant. (**D**) Expression profiles of the candidate genes based on RNA-seq data of PHKs. Column chart was generated based on log2 FPKM (Fragments Per Kilobase Million). Data represent means ± SEM. n = 3.

Supplemental Figure 3

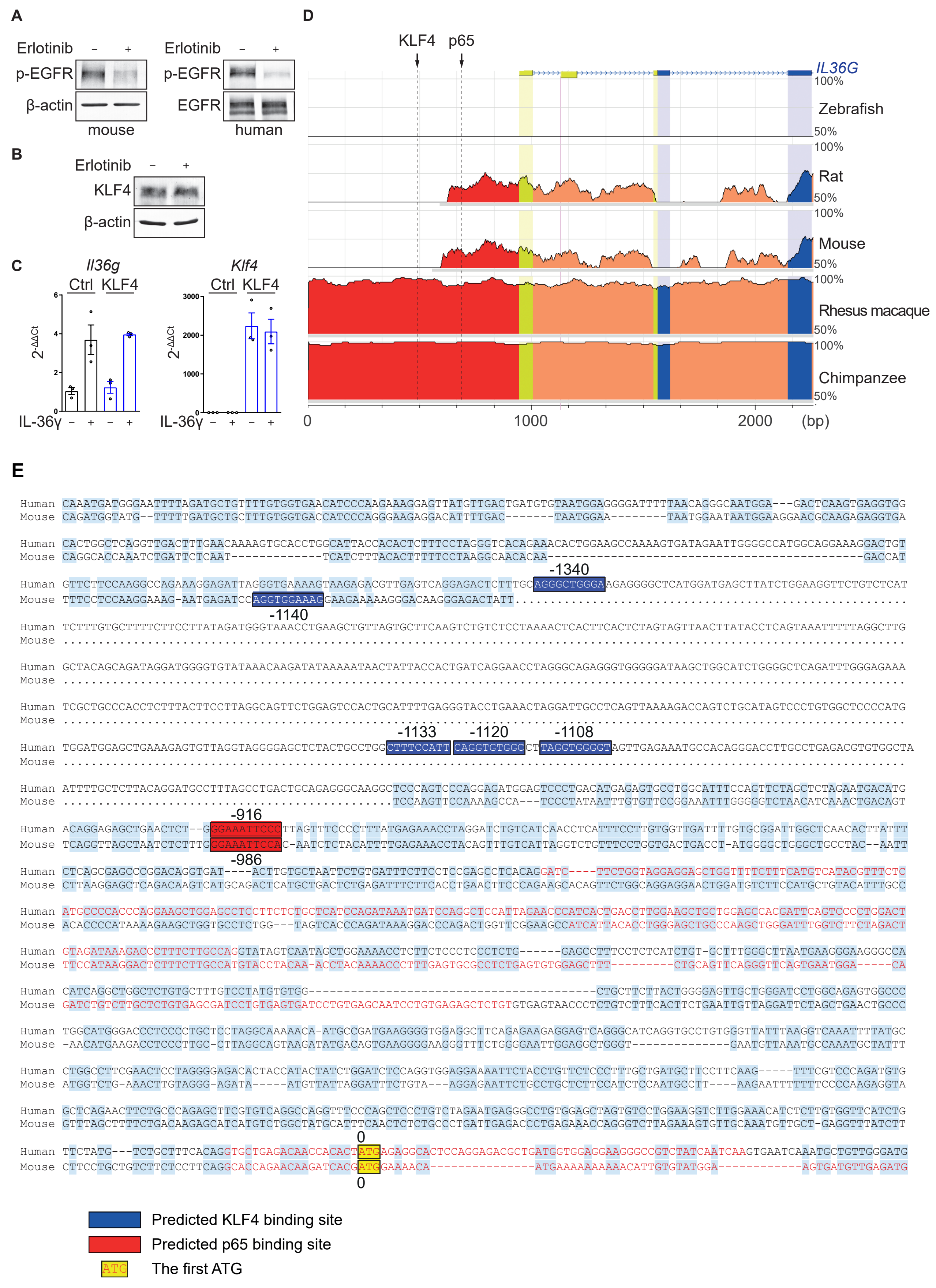

Supplemental Figure 3: Lack of KLF4 binding site results in loss of synergistic IL-36γ production in mice. (**A**) Human and mouse primary keratinocytes were exposed to erlotinib (1 μM) for 1 hour, cell lysates were subjected to SDS-PAGE and immunoblotting. (**B**) PMKs were exposed to erlotinib (1 μM) for 24 hours. Cell lysates were subjected to SDS-PAGE and immunoblotting. All blots were run contemporaneously with the same protein samples. Data are representative of 3 independent experiments. (**C**) KLF4-overexpressing PMKs derived from KLF4-knockout mouse were exposed to mouse IL-36γ (100 ng/mL). Total RNA was subjected to qPCR analysis. Data represent means ± SEM. n = 3. (**D**) Analysis of evolutionarily conserved regions in the genomes of sequenced species referencing to human genome. The y-axis representing % identity between the base and aligned genomes at that specified position. Coding exons are depicted in blue and yellow, blue corresponding to coding exons and yellow to UTRs. Conserved alignments are shown in blue if they overlap with a coding exon and in red or salmon if they correspond to intergenic or intronic regions respectively. (**E**) Alignment of the human IL-36γ gene locus to mouse using Ensembl (35) reveals that the mouse genome lacks the region corresponding to 583 bp long-region of human IL-36γ gene locus including the KLF4-binding site (-1120 bp). The matches between human and mouse sequence are highlighted in blue. The corresponding region to human region (-1591 to -1008 bp) including four putative KLF4-binding sites (-1108, -1120, -1133 and -1340 bp) is missing in the mouse.

Supplemental Figure 4

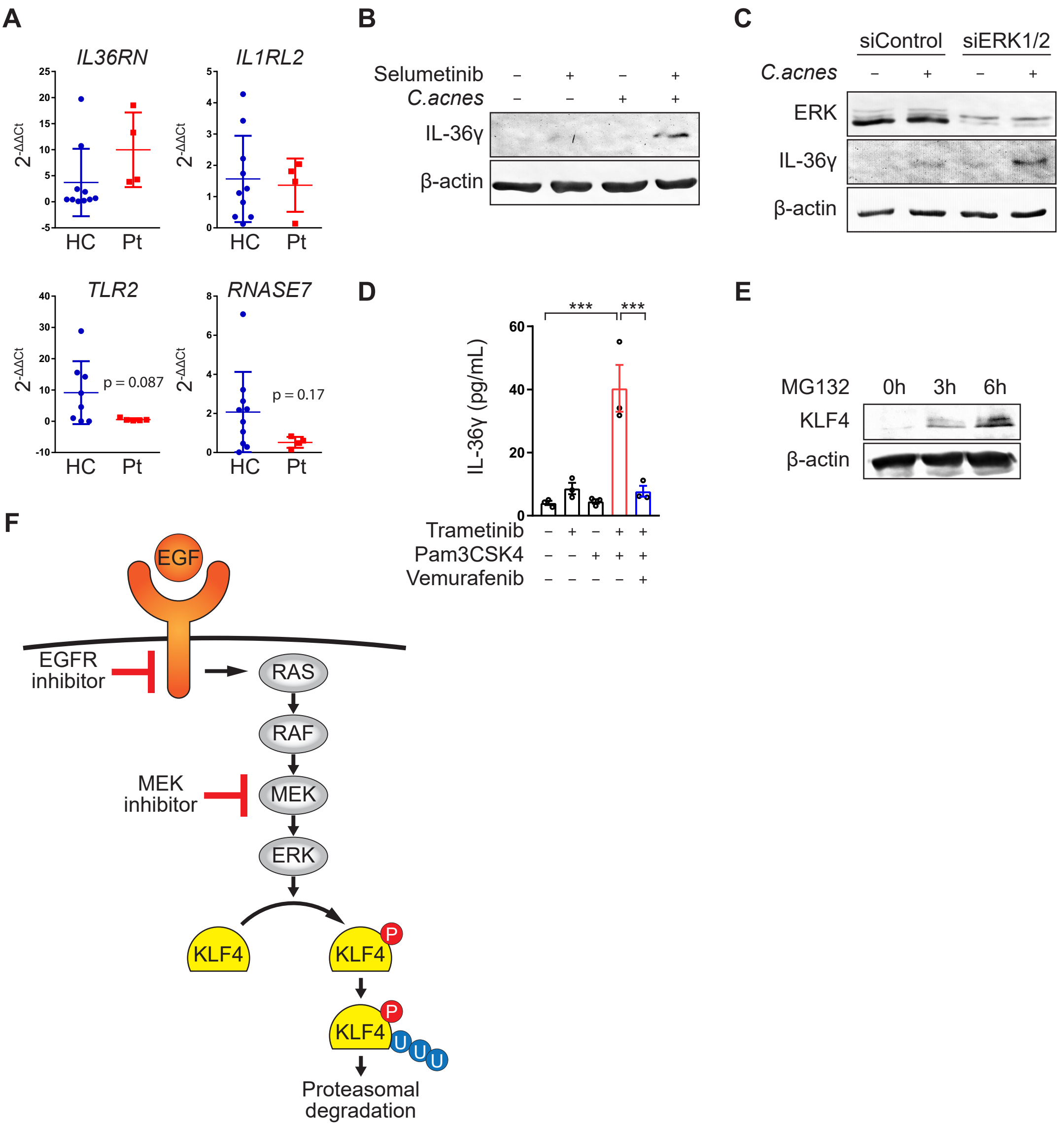

Supplemental Figure 4. Blockade of EGFR-MEK-ERK pathway increases the expression of KLF4 in keratinocytes. (**A**) Quantitative PCR was performed to evaluate gene expression in samples isolated from biopsies of 4 patients with acneiform eruption and 10 healthy control skin biopsies. (**B**) Cell lysates were prepared for western blotting from PHKs exposed to the MEK inhibitor selumetinib (1 μM) and *C. acnes* for 24 hours. (**C**) ERK1/2 siRNA-transduced PHKs were exposed to *C. acnes* for 24 hours. (**D**) PHKs were exposed to trametinib (MEK inhibitor, 2 μg/mL), Pam3CSK4 (5 μg/mL) and vemurafenib (1 μg/mL). The supernatants were analyzed by ELISA. Data represent means ± SEM. n = 3. Data were analyzed with 2-tailed unpaired t test (**A**) or 1-way ANOVA followed by Tukey's multiple-comparisons test (**D**). *P<0.05, **P<0.01, ***P<0.001. (**E**) PHKs were exposed to the proteasome inhibitor MG132 (10 μM) for the indicated time. All blots were run contemporaneously with the same protein samples. Data are representative of 3 independent experiments. (**F**) Schematic of the posttranscriptional regulation of KLF4 by the EGFR-MEK-ERK pathway in keratinocytes. P and U indicate phosphorylation and ubiquitination, respectively.

Supplemental Figure 5

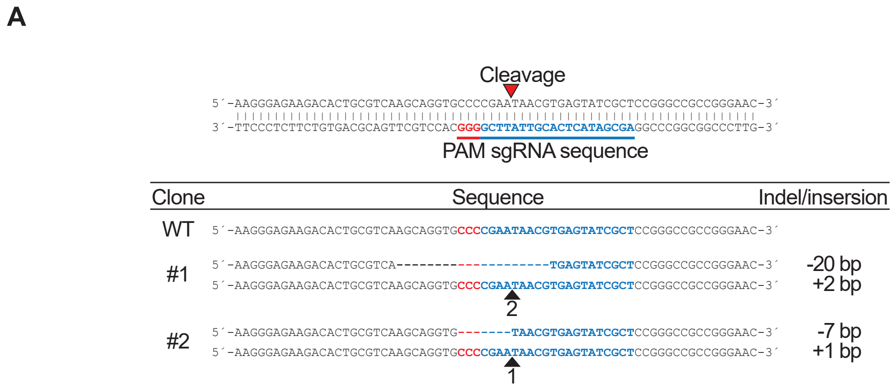

Supplemental Figure 5. The deletion of KLF4 in keratinocyte cell line KERTr cells using the CRISPR/Cas9 system. (A) Mutations generated by CRISPR/Cas9 in exon 2 of KLF4. Red nucleotides are the PAM sequence and blue nucleotides hybridize to the sgRNA.

Supplemental Table 1. Gene expression profiling of lesional skin biopsy samples from patients suffering from acneiform eruption in response to EGFR inhibitor.

Gene Symbol	Gene Name	Fold Change (Log2(EGFRi/HC))	p-value	FDR
ADORA1	Adenosine A1 receptor	-3.302487265	0.315525843	0.556955404
AHSG	Alpha-2-HS-glycoprotein	-1.338487265	0.543273346	0.860557108
AIF1	Allograft inflammatory factor 1	4.651079868	0.122283185	0.362453813
AIMP1	Aminoacyl tRNA synthetase complex-interacting multifunctional protein 1	0.346998239	0.803108921	0.958349861
APCS	Amyloid P component, serum	-3.373887265	0.075537773	0.535484169
APOA2	Apolipoprotein A-II	0.722067471	0.738918017	0.902445466
APOL2	Apolipoprotein L, 2	3.453799661	0.29074965	0.535484169
APOL3	Apolipoprotein L, 3	6.498724216	0.051955123	0.14193768
AREG	Amphiregulin	4.58371944	0.287325271	0.370909506
AZU1	Azurocidin 1	-3.124287265	0.155048679	0.561532807
BCL6	B-cell CLL/lymphoma 6	2.476042813	0.256844233	0.674823684
BLNK	B-cell linker	-0.077332541	0.913877683	0.988097278
BMP1	Bone morphogenetic protein 1	0.318437503	0.955520648	0.958349861
BMP2	Bone morphogenetic protein 2	-1.099203275	0.785235594	0.871147715
BMP3	Bone morphogenetic protein 3	-1.207287265	0.670962846	0.871147715
BMP7	Bone morphogenetic protein 7	0.847652536	0.847882085	0.89375863
C3	Complement component 3	3.812712735	0.218661724	0.512319589
C3AR1	Complement component 3a receptor 1	2.633528781	0.659760023	0.641624526
CARD18	Caspase recruitment domain family, member 18	0.785184329	0.437784488	0.89984875
CAST	Calpastatin	-0.843744809	0.489272999	0.89375863
CCL1	Chemokine (C-C motif) ligand 1	1.522112735	0.61656923	0.84148274
CCL11	Chemokine (C-C motif) ligand 11	-3.595003793	0.098635596	0.535484169
CCL13	Chemokine (C-C motif) ligand 13	1.415879239	0.577814738	0.851454349
CCL16	Chemokine (C-C motif) ligand 16	8.603712735	0.006432949	0.033741679
CCL17	Chemokine (C-C motif) ligand 17	3.422737363	0.2182628	0.535484169
CCL18	Chemokine (C-C motif) ligand 18 (pulmonary and activation-regulated)	0.641189267	0.738761337	0.920779568
CCL19	Chemokine (C-C motif) ligand 19	8.874955777	0.006143627	0.02538234
CCL2	Chemokine (C-C motif) ligand 2	5.079108609	0.076930947	0.302579371
CCL20	Chemokine (C-C motif) ligand 20	10.04224005	0.006164608	0.007015262
CCL21	Chemokine (C-C motif) ligand 21	3.633805557	0.473073703	0.535484169
CCL22	Chemokine (C-C motif) ligand 22	2.655512735	0.228193852	0.641624526
CCL23	Chemokine (C-C motif) ligand 23	-0.265033432	0.820082951	0.959162999
CCL24	Chemokine (C-C motif) ligand 24	3.123312735	0.196869396	0.561532807
CCL25	Chemokine (C-C motif) ligand 25	-1.035887265	0.728729767	0.874441672
CCL26	Chemokine (C-C motif) ligand 26	1.001725943	0.629325883	0.874441672
CCL27	Chemokine (C-C motif) ligand 27	-0.889639313	0.251758528	0.89375863
CCL28	Chemokine (C-C motif) ligand 28	-3.049428348	0.408330375	0.577225302
CCL3	Chemokine (C-C motif) ligand 3	5.629468741	0.144298204	0.236911015
CCL4	Chemokine (C-C motif) ligand 4	0.753213312	0.706532942	0.900177381
CCL5	Chemokine (C-C motif) ligand 5	-3.525535263	0.042915959	0.535484169
CCL7	Chemokine (C-C motif) ligand 7	1.077068411	0.646969362	0.871147715
CCL8	Chemokine (C-C motif) ligand 8	-1.653616879	0.567554934	0.82521587
CCR1	Chemokine (C-C motif) receptor 1	1.484716367	0.656971578	0.842262837
CCR10	Chemokine (C-C motif) receptor 10	-3.373887265	0.075537773	0.535484169
CCR2	Chemokine (C-C motif) receptor 2	-1.168277872	0.733245734	0.871147715
CCR3	Chemokine (C-C motif) receptor 3	1.631312735	0.642106962	0.82521587
CCR4	Chemokine (C-C motif) receptor 4	1.865312735	0.591632504	0.789715183
CCR5	Chemokine (C-C motif) receptor 5	6.189912735	0.083668033	0.172666118
CCR6	Chemokine (C-C motif) receptor 6	4.740312735	0.143258881	0.362453813
CCR7	Chemokine (C-C motif) receptor 7	1.248912735	0.760685666	0.871147715
CCR8	Chemokine (C-C motif) receptor 8	-1.743604813	0.329344077	0.819457327
CCR9	Chemokine (C-C motif) receptor 9	0.434882852	0.867917144	0.950580742
ACKR4	Chemokine (C-C motif) receptor-like 1	10.44611273	0.001429476	0.004882395
CCRL2	Chemokine (C-C motif) receptor-like 2	7.002112735	0.008260908	0.111588847
CD14	CD14 molecule	2.184607077	0.011494375	0.744916351
CD180	CD180 molecule	9.415112735	0.004845117	0.013819557
CD27	CD27 molecule	1.030512735	0.722923861	0.874441672
CD28	CD28 molecule	1.088112735	0.59582379	0.871147715
CD4	CD4 molecule	1.742312735	0.605197129	0.819457327
CD40	CD40 molecule, TNF receptor superfamily member 5	3.903112735	0.437207277	0.507961763
CD40LG	CD40 ligand	3.043574124	0.369754936	0.577225302
CD70	CD70 molecule	-1.627887265	0.589611854	0.82521587
CD74	CD74 molecule, major histocompatibility complex, class II invariant chain	0.279820743	0.809322044	0.959162999
CD86	CD86 molecule	6.404862637	0.047682866	0.148697546
ADGRE5	CD97 molecule	2.038274265	0.216458651	0.775983589
CEBPB	CCAAT/enhancer binding protein (C/EBP), beta	4.635029062	0.202626606	0.362453813
CKLF	Chemokine-like factor	1.947573074	0.417433663	0.778120376
CLC	Charcot-Leyden crystal protein	2.822865945	0.442857498	0.623084257
CMTM1	CKLF-like MARVEL transmembrane domain containing 1	4.545006735	0.192259585	0.378090864
CMTM2	CKLF-like MARVEL transmembrane domain containing 2	-1.177487265	0.648348132	0.871147715
CNTFR	Ciliary neurotrophic factor receptor	1.143512735	0.697855069	0.871147715
CRP	C-reactive protein, pentraxin-related	-1.084487265	0.712705844	0.871147715

CSF1	Colony stimulating factor 1 (macrophage)	0.780470657	0.830612999	0.89984875
CSF2	Colony stimulating factor 2 (granulocyte-macrophage)	-2.637203378	0.412939895	0.641624526
CSF2RA	Colony stimulating factor 2 receptor, alpha, low-affinity (granulocyte-macrophage)	7.631112735	0.01502245	0.079049777
CSF2RB	Colony stimulating factor 2 receptor, beta, low-affinity (granulocyte-macrophage)	2.342180576	0.515883454	0.706160897
CSF3	Colony stimulating factor 3 (granulocyte)	2.518216898	0.318897485	0.666705815
CSF3R	Colony stimulating factor 3 receptor (granulocyte)	4.356112735	0.247044664	0.418841323
CTF1	Cardiotrophin 1	3.766512735	0.367989896	0.519820059
CX3CL1	Chemokine (C-X3-C motif) ligand 1	-1.242689987	0.533463498	0.871147715
CX3CR1	Chemokine (C-X3-C motif) receptor 1	3.236912735	0.185725527	0.561532807
CXCL1	Chemokine (C-X-C motif) ligand 1 (melanoma growth stimulating activity, alpha)	9.376015469	0.012862368	0.013819557
CXCL10	Chemokine (C-X-C motif) ligand 10	-0.537376743	0.822288779	0.92963552
CXCL11	Chemokine (C-X-C motif) ligand 11	-2.514668665	0.610837819	0.666705815
CXCL12	Chemokine (C-X-C motif) ligand 12	-1.228054965	0.29125354	0.871147715
CXCL13	Chemokine (C-X-C motif) ligand 13	5.464451688	0.187383302	0.247507057
CXCL14	Chemokine (C-X-C motif) ligand 14	-0.840810397	0.350548079	0.89375863
CXCL16	Chemokine (C-X-C motif) ligand 16	-0.81450336	0.744566136	0.899788816
CXCL2	Chemokine (C-X-C motif) ligand 2	7.31454686	0.051397524	0.093560865
CXCL3	Chemokine (C-X-C motif) ligand 3	5.283647086	0.240374752	0.272762148
CXCL5	Chemokine (C-X-C motif) ligand 5	5.653332614	0.083198039	0.236911015
CXCL6	Chemokine (C-X-C motif) ligand 6 (granulocyte chemotactic protein 2)	11.53091273	0.000329253	0.001477502
CXCL9	Chemokine (C-X-C motif) ligand 9	1.496385623	0.177874103	0.84148274
CXCR1	Chemokine (C-X-C motif) receptor 1	1.782312735	0.602573099	0.811076575
CXCR2	Chemokine (C-X-C motif) receptor 2	1.551492789	0.611450491	0.840798105
CXCR3	Chemokine (C-X-C motif) receptor 3	-1.222687265	0.573201081	0.871147715
CXCR4	Chemokine (C-X-C motif) receptor 4	5.445512735	0.149275816	0.247507057
CXCR5	Chemokine (C-X-C motif) receptor 5	-3.86058801	0.129902501	0.51170877
CXCR6	Chemokine (C-X-C motif) receptor 6	0.255112735	0.92546011	0.959162999
CYBB	Cytochrome b-245, beta polypeptide	6.186712735	0.067388311	0.172666118
CYP26B1	Cytochrome P450, family 26, subfamily B, polypeptide 1	0.612512735	0.84083183	0.921806758
DOCK2	Dedicator of cytokinesis 2	1.550912735	0.363921258	0.840798105
EBI3	Epstein-Barr virus induced 3	5.963112735	0.082891212	0.201287879
EDA	Ectodysplasin A	3.70729642	0.10575541	0.534957681
EPHX2	Epoxide hydrolase 2, cytoplasmic	-1.556902097	0.602226993	0.840798105
EPO	Erythropoietin	0.875512735	0.755142365	0.89375863
EPOR	Erythropoietin receptor	-1.018487143	0.697197927	0.874441672
ERBB2	V-erb-b2 erythroblastic leukemia viral oncogene homolog 2, neuro/glioblastoma derived oncogene homolog (avian)	6.603912735	0.032446794	0.134090936
ERBB2IP	Erbb2 interacting protein	2.282564737	0.32468204	0.718145721
F11R	F11 receptor	3.548112735	0.240153522	0.535484169
F2	Coagulation factor II (thrombin)	-5.544791043	0.137038846	0.237662687
F3	Coagulation factor III (thromboplastin, tissue factor)	-1.438188507	0.175532538	0.851454349
F8	Coagulation factor VIII, procoagulant component	4.277712735	0.28122353	0.430533761
FASLG	Fas ligand (TNF superfamily, member 6)	1.072387338	0.79492053	0.871147715
FGF1	Fibroblast growth factor 1 (acidic)	1.825312735	0.587574529	0.798067292
FGF10	Fibroblast growth factor 10	-4.013687265	0.098627783	0.487605184
FGF12	Fibroblast growth factor 12	4.106312735	0.218501167	0.464791464
FGF2	Fibroblast growth factor 2 (basic)	0.288320053	0.903202644	0.959162999
FGF7	Fibroblast growth factor 7	-0.405171243	0.741681008	0.950580742
FIGF	C-fos induced growth factor (vascular endothelial growth factor D)	-3.699915091	0.048791736	0.534957681
FLT3LG	Fms-related tyrosine kinase 3 ligand	5.217312735	0.095357088	0.279142589
FN1	Fibronectin 1	1.1735793	0.093756018	0.871147715
FOS	FBJ murine osteosarcoma viral oncogene homolog	-0.260739426	0.894445981	0.959162999
FPR1	Formyl peptide receptor 1	4.471512735	0.267244734	0.387256035
GDF2	Growth differentiation factor 2	-2.060487265	0.265693065	0.775105108
GDF3	Growth differentiation factor 3	-3.373887265	0.075537773	0.535484169
GDF5	Growth differentiation factor 5	-4.527863705	0.099919291	0.378894973
GDF6	Growth differentiation factor 6	-3.373887265	0.075537773	0.535484169
GDF9	Growth differentiation factor 9	4.656887509	0.054033321	0.362453813
GFRA1	GDNF family receptor alpha 1	5.003512735	0.034573893	0.318170266
GFRA2	GDNF family receptor alpha 2	5.189112735	0.135189668	0.279142589
GHR	Growth hormone receptor	0.900589902	0.544570309	0.893612279
GLMN	Glomulin, FKBP associated protein	3.850030179	0.275582225	0.51170877
GPI	Glucose-6-phosphate isomerase	-0.909462607	0.317790313	0.893488874
GPR68	G protein-coupled receptor 68	2.628312735	0.456487103	0.641624526
GREM1	Gremlin 1	2.084792441	0.687268471	0.772766497
GREM2	Gremlin 2	5.205146725	0.139945317	0.279142589
GRN	Granulin	3.506363339	0.061751578	0.535484169
HDAC4	Histone deacetylase 4	3.661912735	0.227570526	0.535484169
HDAC5	Histone deacetylase 5	7.648112735	0.066872452	0.079049777
HDAC7	Histone deacetylase 7	0.093843576	0.967843123	0.988097278
HDAC9	Histone deacetylase 9	-2.050607077	0.458363567	0.775105108
HRH1	Histamine receptor H1	7.009312735	0.018016177	0.111588847

IFNA1	Interferon, alpha 1	-5.729206998	0.152544453	0.231778967
IFNA14	Interferon, alpha 14	-7.298836392	0.044735624	0.093560865
IFNA2	Interferon, alpha 2	-5.815087265	0.103551834	0.218024462
IFNA4	Interferon, alpha 4	-3.373887265	0.075537773	0.535484169
IFNA8	Interferon, alpha 8	-1.590853867	0.586860864	0.835855432
IFNAR1	Interferon (alpha, beta and omega) receptor 1	4.707484085	0.153532614	0.362453813
IFNAR2	Interferon (alpha, beta and omega) receptor 2	4.78175289	0.142324143	0.360936469
IFNB1	Interferon, beta 1, fibroblast	-1.433887265	0.681206168	0.851454349
IFNE	Interferon, epsilon	-0.855287265	0.785672012	0.89375863
IFNG	Interferon, gamma	1.122455502	0.743624467	0.871147715
IFNGR1	Interferon gamma receptor 1	3.455486026	0.140949705	0.535484169
IFNGR2	Interferon gamma receptor 2 (interferon gamma transducer 1)	0.409428149	0.789319585	0.950580742
IFNK	Interferon, kappa	1.981512735	0.575127501	0.77615293
IFNW1	Interferon, omega 1	-3.373887265	0.075537773	0.535484169
IFNWP2	Interferon, omega 1 pseudogene 2	-0.984287265	0.746723796	0.876502114
IK	IK cytokine, down-regulator of HLA II	6.415826889	0.127382445	0.148697546
IL10	Interleukin 10	5.454312735	0.137230289	0.247507057
IL10RA	Interleukin 10 receptor, alpha	0.528543317	0.672574956	0.929733525
IL10RB	Interleukin 10 receptor, beta	3.131837326	0.13259828	0.561532807
IL11	Interleukin 11	0.213712735	0.919674167	0.969262724
IL11RA	Interleukin 11 receptor, alpha	-2.723048666	0.556478275	0.641110458
IL12A	Interleukin 12A (natural killer cell stimulatory factor 1, cytotoxic lymphocyte maturation factor 1, p35)	4.770510178	0.38071035	0.360936469
IL12B	Interleukin 12B (natural killer cell stimulatory factor 2, cytotoxic lymphocyte maturation factor 2, p40)	-3.412487265	0.076565641	0.535484169
IL12RB1	Interleukin 12 receptor, beta 1	0.786712735	0.822375892	0.89984875
IL12RB2	Interleukin 12 receptor, beta 2	1.865912735	0.584754952	0.789715183
IL13	Interleukin 13	-3.861955936	0.173372275	0.51170877
IL13RA1	Interleukin 13 receptor, alpha 1	5.29387081	0.040144645	0.272762148
IL13RA2	Interleukin 13 receptor, alpha 2	4.6272215	0.431485875	0.362453813
IL15	Interleukin 15	0.122612991	0.935679251	0.988097278
IL15RA	Interleukin 15 receptor, alpha	2.732877853	0.441551401	0.641110458
IL16	Interleukin 16	0.323512735	0.924413272	0.958349861
IL17A	Interleukin 17A	-4.015887265	0.011866652	0.487605184
IL17B	Interleukin 17B	-3.129421115	0.452143773	0.561532807
IL17C	Interleukin 17C	-1.941129532	0.654551814	0.778120376
IL17D	Interleukin 17D	-1.507884427	0.590610624	0.84148274
IL17F	Interleukin 17F	4.627512735	0.193766394	0.362453813
IL17RA	Interleukin 17 receptor A	-2.540247177	0.323296684	0.666705815
IL17RB	Interleukin 17 receptor B	-2.207897836	0.631337672	0.74317631
IL18	Interleukin 18 (interferon-gamma-inducing factor)	-0.425817108	0.689343326	0.950580742
IL18R1	Interleukin 18 receptor 1	3.917512735	0.24466944	0.507961763
IL18RAP	Interleukin 18 receptor accessory protein	4.300512735	0.174299765	0.427680613
IL19	Interleukin 19	8.273712735	0.00498753	0.045096857
IL1A	Interleukin 1, alpha	1.097585715	0.767611698	0.871147715
IL1B	Interleukin 1, beta	7.023573605	0.093326459	0.111588847
IL1F10	Interleukin 1 family, member 10 (theta)	2.513112735	0.479680321	0.666705815
IL1R1	Interleukin 1 receptor, type I	1.986522525	0.704989055	0.77615293
IL1R2	Interleukin 1 receptor, type II	-0.617141757	0.643421185	0.921806758
IL1RAP	Interleukin 1 receptor accessory protein	3.176203763	0.267761604	0.561532807
IL1RAPL2	Interleukin 1 receptor accessory protein-like 2	-5.613640988	0.139459736	0.236911015
IL1RL1	Interleukin 1 receptor-like 1	7.760575095	0.063958529	0.075316269
IL1RL2	Interleukin 1 receptor-like 2	3.177712735	0.420457986	0.561532807
IL1RN	Interleukin 1 receptor antagonist	-1.428520392	0.223670941	0.851454349
IL2	Interleukin 2	-3.262351944	0.385108593	0.561532807
IL20	Interleukin 20	-1.244799844	0.307914979	0.871147715
IL20RA	Interleukin 20 receptor, alpha	3.285955118	0.305370705	0.559026448
IL21	Interleukin 21	-3.373887265	0.075537773	0.535484169
IL21R	Interleukin 21 receptor	0.403596402	0.904330959	0.950580742
IL22	Interleukin 22	-2.71124581	0.124533441	0.641110458
IL22RA1	Interleukin 22 receptor, alpha 1	-2.094022421	0.06381785	0.772766497
IL22RA2	Interleukin 22 receptor, alpha 2	3.794732474	0.128781951	0.51420204
IL23A	Interleukin 23, alpha subunit p19	1.345712735	0.66589506	0.860557108
IL23R	Interleukin 23 receptor	-2.748026718	0.484935204	0.641110458
IL24	Interleukin 24	10.21679207	0.002901162	0.006069247
IL25	Interleukin 25	-3.052479636	0.384752713	0.577225302
IL26	Interleukin 26	4.129312735	0.147910881	0.461608668
IL27	Interleukin 27	-0.343287265	0.921773197	0.958349861
IFNLR1	Interleukin 28 receptor, alpha (interferon, lambda receptor)	-0.193287265	0.94202	0.97285991
IFNL1	Interleukin 29 (interferon, lambda 1)	7.292712735	0.013932919	0.093560865
IL2RA	Interleukin 2 receptor, alpha	1.950587448	0.312639888	0.778120376
IL2RB	Interleukin 2 receptor, beta	-3.373887265	0.075537773	0.535484169
IL2RG	Interleukin 2 receptor, gamma	3.833112735	0.31313083	0.51170877
IL3	Interleukin 3 (colony-stimulating factor, multiple)	-3.373887265	0.075537773	0.535484169
IL31RA	Interleukin 31 receptor A	-3.373887265	0.075537773	0.535484169
IL32	Interleukin 32	3.03883074	0.056711274	0.577225302
IL36A	Interleukin 36, alpha	-1.631934378	0.679400206	0.82521587

IL36B	Interleukin 36, beta	-2.348141129	0.504913319	0.706160897
IL36G	Interleukin 36, gamma	10.77747558	0.00275906	0.003387827
IL36RN	Interleukin 36 receptor antagonist	3.154349191	0.32233765	0.561532807
IL37	Interleukin 37	-1.255901855	0.350301804	0.871147715
IL3RA	Interleukin 3 receptor, alpha (low affinity)	0.498424191	0.899975938	0.936707868
IL4	Interleukin 4	0.998712735	0.807637916	0.874441672
IL4R	Interleukin 4 receptor	2.029658878	0.511037617	0.775983589
IL5	Interleukin 5 (colony-stimulating factor, eosinophil)	1.636512735	0.618517438	0.82521587
IL5RA	Interleukin 5 receptor, alpha	-3.907322861	0.44772166	0.507961763
IL6	Interleukin 6 (interferon, beta 2)	2.805095331	0.530228589	0.626011427
IL6R	Interleukin 6 receptor	4.648112735	0.147425237	0.362453813
IL6ST	Interleukin 6 signal transducer (gp130, oncostatin M receptor)	3.128157901	0.130621276	0.561532807
IL7	Interleukin 7	5.996312735	0.139676959	0.199152959
IL7R	Interleukin 7 receptor	7.910712735	0.007926847	0.066543269
CXCL8	Interleukin 8	12.79871273	0.001119637	0.000624233
IL9	Interleukin 9	-0.965322073	0.766695759	0.880118454
IL9R	Interleukin 9 receptor	-6.644006168	0.109736981	0.134090936
INHA	Inhibin, alpha	-3.373887265	0.075537773	0.535484169
INHBA	Inhibin, beta A	1.415905759	0.67311827	0.851454349
INHBB	Inhibin, beta B	0.661512735	0.804280053	0.916876697
INS	Insulin	-1.660663504	0.633143724	0.82521587
IRF4	Interferon regulatory factor 4	-2.319744351	0.613520487	0.7087413
IRF7	Interferon regulatory factor 7	3.129912735	0.214099701	0.561532807
ITGB2	Integrin, beta 2 (complement component 3 receptor 3 and 4 subunit)	7.508712735	0.068829677	0.086891278
ITIH4	Inter-alpha (globulin) inhibitor H4 (plasma Kallikrein-sensitive glycoprotein)	4.238712735	0.13969986	0.438766185
KITLG	KIT ligand	2.938558713	0.241518784	0.592815575
KNG1	Kininogen 1	0.16547634	0.9719696	0.978710586
LBP	Lipopolysaccharide binding protein	-0.568287265	0.819603071	0.925182556
LEFTY1	Left-right determination factor 1	-1.495655472	0.623728285	0.84148274
LEFTY2	Left-right determination factor 2	-1.682501236	0.604909022	0.82521587
LEPR	Leptin receptor	0.581018039	0.91526413	0.925182556
LIF	Leukemia inhibitory factor (cholinergic differentiation factor)	2.316712735	0.533078074	0.7087413
LIFR	Leukemia inhibitory factor receptor alpha	1.996696091	0.726167331	0.77615293
LTA	Lymphotoxin alpha (TNF superfamily, member 1)	-3.373887265	0.075537773	0.535484169
LTB	Lymphotoxin beta (TNF superfamily, member 3)	-0.036093497	0.991666158	0.993749543
LTB4R	Leukotriene B4 receptor	-0.109928369	0.898743076	0.988097278
LY75	Lymphocyte antigen 75	5.578362796	0.094448759	0.236911015
LY86	Lymphocyte antigen 86	4.823912735	0.168296179	0.354764832
LY96	Lymphocyte antigen 96	0.329596158	0.831029828	0.958349861
MDK	Midkine (neurite growth-promoting factor 2)	1.341495444	0.3701504	0.860557108
MEFV	Mediterranean fever	3.455112735	0.336111173	0.535484169
MGLL	Monoglyceride lipase	-0.78744292	0.260929795	0.89984875
MIF	Macrophage migration inhibitory factor (glycosylation-inhibiting factor)	-0.374182788	0.803283822	0.956045236
MMP25	Matrix metallopeptidase 25	-0.072687265	0.974243918	0.988097278
MPL	Myeloproliferative leukemia virus oncogene	1.174912735	0.694291776	0.871147715
MSTN	Myostatin	-2.588560739	0.608519237	0.652724592
MUC4	Mucin 4, cell surface associated	1.174912735	0.692972196	0.871147715
MYD88	Myeloid differentiation primary response gene (88)	0.36912991	0.466401477	0.956045236
NAMPT	Nicotinamide phosphoribosyltransferase	3.618774924	0.135773681	0.535484169
NCR3	Natural cytotoxicity triggering receptor 3	-3.373887265	0.075537773	0.535484169
NFAM1	NFAT activating protein with ITAM motif 1	2.157312735	0.54990435	0.751843146
NFATC3	Nuclear factor of activated T-cells, cytoplasmic, calcineurin-dependent 3	-0.305655112	0.826710993	0.959162999
NFATC4	Nuclear factor of activated T-cells, cytoplasmic, calcineurin-dependent 4	3.181597006	0.213866892	0.561532807
NFE2L1	Nuclear factor (erythroid-derived 2)-like 1	2.051312735	0.461008493	0.775105108
NFKB1	Nuclear factor of kappa light polypeptide gene enhancer in B-cells 1	1.367444315	0.501052459	0.860557108
NFRKB	Nuclear factor related to kappaB binding protein	2.706912735	0.25526592	0.641110458
NFX1	Nuclear transcription factor, X-box binding 1	4.142514218	0.171095747	0.461608668
NLRP12	NLR family, pyrin domain containing 12	0.932712735	0.742414968	0.888417366
NMI	N-myc (and STAT) interactor	-0.568131464	0.543864861	0.925182556
NODAL	Nodal homolog (mouse)	-1.081687265	0.713636871	0.871147715
NOS2	Nitric oxide synthase 2, inducible	-3.155033884	0.552014046	0.561532807
NOX5	NADPH oxidase, EF-hand calcium binding domain 5	-1.119038858	0.69712	0.871147715
NR3C1	Nuclear receptor subfamily 3, group C, member 1 (glucocorticoid receptor)	4.138611063	0.295587948	0.461608668
OLR1	Oxidized low density lipoprotein (lectin-like) receptor 1	2.926112735	0.170730616	0.592815575
OSM	Oncostatin M	3.527112735	0.398731024	0.535484169
OSMR	Oncostatin M receptor	3.190866116	0.185480145	0.561532807
PARP4	Poly (ADP-ribose) polymerase family, member 4	6.500595505	0.04771622	0.14193768
PDGFA	Platelet-derived growth factor alpha polypeptide	0.750413748	0.697685357	0.900177381
PDGFB	Platelet-derived growth factor beta polypeptide	-1.519274728	0.625355165	0.84148274
PF4V1	Platelet factor 4 variant 1	-2.677202505	0.542026677	0.641624526

PGLYRP1	Peptidoglycan recognition protein 1	1.153312735	0.703370301	0.871147715
PLA2G2D	Phospholipase A2, group IID	-6.227030905	0.267544393	0.172666118
PLA2G7	Phospholipase A2, group VII (platelet-activating factor acetylhydrolase, plasma)	1.524238196	0.488043496	0.84148274
PPBP	Pro-platelet basic protein (chemokine (C-X-C motif) ligand 7)	-0.262789157	0.950098419	0.959162999
PRDX5	Peroxiredoxin 5	1.067450113	0.210217581	0.871147715
PREX1	Phosphatidylinositol-3,4,5-trisphosphate-dependent Rac exchange factor 1	2.664512735	0.28594418	0.641624526
PRG2	Proteoglycan 2, bone marrow (natural killer cell activator, eosinophil granule major basic protein)	1.6562819	0.735859812	0.82521587
PRG3	Proteoglycan 3	-3.373887265	0.075537773	0.535484169
PRL	Prolactin	-1.124487265	0.682929849	0.871147715
PRLR	Prolactin receptor	5.597112735	0.123420663	0.236911015
PROCR	Protein C receptor, endothelial	2.420838474	0.49221821	0.690624646
PROK2	Prokineticin 2	2.994658847	0.483073783	0.586480661
PTAFR	Platelet-activating factor receptor	7.170136484	0.043034751	0.103300226
PTGS2	Prostaglandin-endoperoxide synthase 2 (prostaglandin G/H synthase and cyclooxygenase)	1.01365181	0.841388089	0.874441672
PTN	Pleiotrophin	1.927611295	0.370809118	0.779804349
PTPRA	Protein tyrosine phosphatase, receptor type, A	2.849912735	0.343104616	0.616775106
PTX3	Pentraxin 3, long	12.32331273	0.002576993	0.000743371
PXMP2	Peroxisomal membrane protein 2, 22kDa	1.901205582	0.390168864	0.786422861
REG3A	Regenerating islet-derived 3 alpha	-4.850487265	0.134364801	0.354764832
REG3G	Regenerating islet-derived 3 gamma	-6.632992477	0.117638812	0.134090936
RIPK2	Receptor-interacting serine-threonine kinase 2	-0.107327585	0.890767935	0.988097278
S100A12	S100 calcium binding protein A12	11.81679177	0.00458345	0.0012089
S100A8	S100 calcium binding protein A8	10.81374342	0.00454918	0.003387827
S100B	S100 calcium binding protein B	-6.612329752	0.064707934	0.134090936
SAA4	Serum amyloid A4, constitutive	0.855729898	0.685830036	0.89375863
SCUBE1	Signal peptide, CUB domain, EGF-like 1	-1.161887265	0.686599165	0.871147715
SDCBP	Syndecan binding protein (syntenin)	2.940533377	0.232379017	0.592815575
SECTM1	Secreted and transmembrane 1	-0.745750815	0.841732181	0.900177381
SELE	Selectin E	6.764312735	0.110026789	0.126878221
SERPINA1	Serpin peptidase inhibitor, clade A (alpha-1 antiproteinase, antitrypsin), member 1	6.929312735	0.079372696	0.113042406
SERPINA3	Serpin peptidase inhibitor, clade A (alpha-1 antiproteinase, antitrypsin), member 3	6.895312735	0.140950703	0.113633
SERPINF2	Serpin peptidase inhibitor, clade F (alpha-2 antiplasmin, pigment epithelium derived factor), member 2	2.672312735	0.198310825	0.641624526
SFTPD	Surfactant protein D	2.003699472	0.569740482	0.77615293
SIGIRR	Single immunoglobulin and toll-interleukin 1 receptor (TIR) domain	-2.705803629	0.461927933	0.641110458
SIGLEC1	Sialic acid binding Ig-like lectin 1, sialoadhesin	-0.085155399	0.979843645	0.988097278
SLCO1A2	Solute carrier organic anion transporter family, member 1A2	-3.922677512	0.494676792	0.507961763
SLURP1	Secreted LY6/PLAUR domain containing 1	1.014025928	0.598228825	0.874441672
SOCS2	Suppressor of cytokine signaling 2	6.121886404	0.141840317	0.180030134
SPACA3	Sperm acrosome associated 3	-3.373887265	0.075537773	0.535484169
SPP1	Secreted phosphoprotein 1	9.492608774	0.064520722	0.013819557
SPRED1	Sprouty-related, EVH1 domain containing 1	5.912288028	0.138950084	0.206988092
SRGAP1	SLIT-ROBO Rho GTPase activating protein 1	-1.690973386	0.682878026	0.82521587
STAB1	Stabilin 1	1.696112735	0.666786808	0.82521587
STAT3	Signal transducer and activator of transcription 3 (acute-phase response factor)	3.827247485	0.143479892	0.51170877
SYK	Spleen tyrosine kinase	6.962493851	0.043207184	0.11265625
TACR1	Tachykinin receptor 1	0.667204141	0.894751273	0.916876697
THPO	Thrombopoietin	-5.860252621	0.324798168	0.213119192
TIRAP	Toll-interleukin 1 receptor (TIR) domain containing adaptor protein	1.973712735	0.570375854	0.77615293
TLR1	Toll-like receptor 1	5.566912735	0.050096295	0.236911015
TLR10	Toll-like receptor 10	0.543237888	0.838516353	0.92963552
TLR2	Toll-like receptor 2	2.363312735	0.354868554	0.705252741
TLR3	Toll-like receptor 3	1.352930081	0.566713542	0.860557108
TLR4	Toll-like receptor 4	2.935157687	0.364565497	0.592815575
TLR5	Toll-like receptor 5	8.280912735	0.014881605	0.045096857
TLR6	Toll-like receptor 6	4.649512735	0.033465861	0.362453813
TLR7	Toll-like receptor 7	0.038640024	0.961458487	0.993749543
TLR8	Toll-like receptor 8	2.663912735	0.482658647	0.641624526
TLR9	Toll-like receptor 9	-1.078087265	0.714832507	0.871147715
TNF	Tumor necrosis factor	2.472512735	0.416127298	0.674823684
TNFAIP6	Tumor necrosis factor, alpha-induced protein 6	2.368277615	0.399280785	0.705252741
TNFRSF11B	Tumor necrosis factor receptor superfamily, member 11b	1.986757394	0.392891517	0.77615293
TNFSF10	Tumor necrosis factor (ligand) superfamily, member 10	3.182543381	0.182643546	0.561532807
TNFSF11	Tumor necrosis factor (ligand) superfamily, member 11	-4.479526633	0.099757406	0.387256035
TNFSF13	Tumor necrosis factor (ligand) superfamily, member 13	-2.982970596	0.08746913	0.586520505
TNFSF13B	Tumor necrosis factor (ligand) superfamily, member 13b	5.59497446	0.088991473	0.236911015
TNFSF14	Tumor necrosis factor (ligand) superfamily, member 14	0.728712735	0.833673956	0.902445466
TNFSF15	Tumor necrosis factor (ligand) superfamily, member 15	1.382512735	0.660299352	0.860557108

TNFSF18	Tumor necrosis factor (ligand) superfamily, member 18	-1.112190414	0.696211609	0.871147715
TNFSF4	Tumor necrosis factor (ligand) superfamily, member 4	4.341967178	0.126885178	0.418841323
TNFSF8	Tumor necrosis factor (ligand) superfamily, member 8	1.891112735	0.596452113	0.786799314
TNFSF9	Tumor necrosis factor (ligand) superfamily, member 9	-2.196689664	0.119434017	0.74387357
TOLLIP	Toll interacting protein	5.206512735	0.232749405	0.279142589
TPST1	Tyrosylprotein sulfotransferase 1	7.458712735	0.00484763	0.087895572
TRAP1	TNF receptor-associated protein 1	-1.724960934	0.529199593	0.822629445
TTN	Titin	-0.594229178	0.845173135	0.925007813
TYMP	Thymidine phosphorylase	4.828234732	0.057405232	0.354764832
VEGFA	Vascular endothelial growth factor A	0.472933377	0.690490672	0.942167111
VEGFB	Vascular endothelial growth factor B	1.834568137	0.382730825	0.79800134
VPS45	Vacuolar protein sorting 45 homolog (S. cerevisiae)	-0.769633444	0.570609957	0.900177381
XCL1	Chemokine (C motif) ligand 1	2.992962747	0.590964635	0.586480661
XCR1	Chemokine (C motif) receptor 1	0.613196127	0.857269814	0.921806758
YARS	Tyrosyl-tRNA synthetase	5.327202365	0.180102257	0.270781622

Supplemental Table 2. The primers used for amplification of specific genes in this study.

Gene	Forward primer	Reverse primer
Human		
ARNT	5'-TGA AGC CGC CAT CTT GGA TT-3'	5'-GGT CCA GAG TTT CCA GAG GC-3'
bHLHE40	5'-AGC AGT GGT TCT TGA ACT TAC C-3'	5'-ACA AGC TGC GAA GAC TTC AGG-3'
CCL20	5`-AAG TTG TCT GTG TGC GCA AAT CC-3'	5`-CCA TTC CAG AAA AGC CAC AGT TTT-3'
CXCL1	5'-GCC CAA ACC GAA GTC ATA GCC-3'	5'-TAA CTA TGG GGG ATG CAG GA-3'
CXCL6	5'-AGC GCT GGT CCT GTC TCT G-3'	5'-CTC AGC GTA ACG CGT AAA CA-3'
EN1	5'-CGT GGT CAA AAC TGA CTC GC-3'	5'-CTT GAG TCT CTG CAG CTG CT-3'
IL17	5`-CAA TCC CAC GAA ATC CAG GAT G-3'	5`-GGT GGA GAT TCC AAG GTG AGG-3'
IL1β	5`-CAC GAT GCA CCT GTA CGA TCA-3'	5`-GTT GCT CCA TAT CCT GTC CCT-3'
IL24	5'-TGT GAA AGA CAC TAT GCA AGC TC-3'	5'-GTG ACA CGA TGA GAA CAA AGT TG-3'
IL36	5`-CCA GAC GCT CAT AGC AGT CC-3'	5`-AGA TGG GGT TCC CTC TGT CTT-3'
IL36R	5`-CCG AGG TGT TGG AGA GAC AAT G-3'	5`-GGA CCA CAA TGA CAA TCA GCC TC-3'
IL36Ra	5`-ACT CGG CAT TGA AGG TGC TTT-3'	5`-GGG ACC ACG CTG ATC TCT T-3'
IL36β	5`-ATG AAC CCA CAA CGG GAG G-3`	5`-TAA TGC TGC GGC TAA GAG GAG-3`
IL36γ	5`-AGG AAG GGC CGT CTA TCA ATC-3'	5`-CAC TGT CAC TTC GTG GAA CTG-3'
IL6	5`-AAA TTC GGT ACA TCC TCG ACG G-3'	5`-GGA AGG TTC AGG TTG TTT TCT GC-3'
IL8	5`-TTT TGC CAA GGA GTG CTA AAG A-3'	5`-AAC CCT CTG CAC CCA GTT TTC-3'
KLF1	5'-CCG GAC ACA CAG GAT GAC TT-3'	5'-CCG AGA AGT TGG TGA GGA GG-3'
KLF4	5'-CCCACATGAAGCGACTTCCC-3'	5'-CAGGTCCAGGAGATCGTTGAA-3'
KLF5	5'-CCT GGT CCA GAC AAG ATG TGA-3'	5'-GAA CTG GTC TAC GAC TGA GGC-3'
MYOD1	5'-ATC CGC TAT ATC GAG GGC CT-3'	5'-ATC CGC TAT ATC GAG GGC CT-3'
MYOG	5'-TGC CAT CCA GTA CAT CGA GC-3'	5'-GGG CAT GGT TTC ATC TGG GA-3'
RNase7	5'-CGT GTC CCT GAC CAT GTG TAA-3'	5'-GAC TTG TTC TGT CGC TTC TCT T-3'
RUNX1	5'-TCA GTG AAC TGG AGC AGC TG-3'	5'-CTG GGT GCA CAG AAG GAG AG-3'
RUNX2	5'-CGG AGA GGT ACC AGA TGG GA-3'	5'-CCG GCC CAC AAA TCT CAG AT-3'
S100A12	5'-AGC ATC TGG AGG GAA TTG TCA-3'	5'-GCA ATG GCT ACC AGG GAT ATG AA-3'
S100A8	5`-ATG CCG TCT ACA GGG ATG AC-3'	5`-ACA CTC GGT CTC TAG CAA TTT CT-3'
TCF12	5'-AGT TAT CCA TCT CCT AAG CCA CC-3'	5'-AAG AAT TGT GGG TCC CAT CTT G-3'
TLR2	5`-CTT CAC TCA GGA GCA GCA AGC A-3'	5`-ACA CCA GTG CTG TCC TGT GAC A-3'
TNFα	5`-ATG AGC ACT GAA AGC ATG ATC C-3'	5`-GAG GGC TGA TTA GAG AGA GGT C-3'
ZEB1	5'-CAC TGC CCA GTT ACC CAC AA-3'	5'-CAG GGC TGA CCG TAG TTG AG-3'
ZFX	5'-TGC AAA CAA CTC GAG CTG GA-3'	5'-ACC ATC AGC TCC TGT TGC AT-3'
Mouse		
IL36γ	5'-CAG GTG TGG ATC TTT CGT AAT CA-3'	5'-CAT GGG AGG ATA GTC ACG CTG-3'
KLF4	5'-GTG CCC CGA CTA ACC GTT-3'	5'-CTA GGT CCA GGA GGT CGT-3'

Supplemental Table 3. The sgRNAs used in this study.

sgRNAs	Sequence (5' - 3')
human KLF4-F	caccgAGCGATACTCACGTTATTCG
human KLF4-R	aaacCGAATAACGTGAGTATCGCTc
KLF4 binding site on human IL36g promoter-F	caccgCACCTAAGGCCACACCTGAA
KLF4 binding site on human IL36g promoter-R	aaacTTCAGGTGTGGCCTTAGGTGc
Control-F	caccgGGCCGATAATGATCCGACCG
Control-R	aaacCGGTCGGATCATTATCGGCCc
